# Glutamate-induced nuclear translocation of PYK2 in hippocampal neurons, interaction with MBD2, and role in cell death in a model of epilepsy

**DOI:** 10.1038/s41419-026-08628-x

**Published:** 2026-04-22

**Authors:** Albert Giralt, Tiago Mendes, Enrica Montalban, Carmen Cifuentes-Diaz, Marcos Galán-Ganga, Margot Chouchana, Benoît de Pins, Sophie Longueville, Damien Carrel, Jean-Antoine Girault

**Affiliations:** 1 Inserm UMR-S 1270, Paris, France; 2 Sorbonne Université, Faculté des Sciences et d’Ingénierie, Paris, France; 3 Institut du Fer à Moulin, Paris, France; 4 Departament de Biomedicina, Facultat de Medicina i Ciències de la Salut, Institut de Neurociències, Universitat de Barcelona, Barcelona, Spain; 5 Centro de Investigación Biomédica en Red sobre Enfermedades Neurodegenerativas (CIBERNED), Madrid, Spain; 6 Institut d’Investigacions Biomèdiques August Pi i Sunyer (IDIBAPS), Barcelona, Spain; 7 Production and Validation Center of Advanced Therapies (Creatio), University of Barcelona, Barcelona, Spain; 8 Present Address: Astrazeneka, Göteborg, Sweden; 9 Present Address: Université de Bordeaux, INRAE, UMR 1286, Bordeaux, France; 10 Present Address: Université Paris Cité, Inserm, UMRS-1144, Faculté de pharmacie de Paris, Paris, France; 11 Present Address: Department of Biology, University of Naples “Federico II”, Naples, Italy; 12 Present Address: Université de Paris, Centre National de la Recherche Scientifi que (CNRS), UMR 8003, Paris, France; 13 Present Address: Institut du Cerveau (ICM), Paris Brain Institute, Inserm, CNRS, Sorbonne Université, Paris, France

**Keywords:** Cell death in the nervous system, Mechanisms of disease

## Abstract

Synaptic activity results in long-lasting alterations of neuronal properties, which require gene expression regulation. PYK2 is a calcium-activated non-receptor protein tyrosine kinase highly expressed in hippocampal neurons and involved in synaptic functions. PYK2 also shuttles between the nucleus and the cytoplasm. We show that glutamate stimulation induces PYK2 accumulation in the nucleus of hippocampal neurons in culture through activation of NMDA receptors, L-type voltage-gated Ca^2+^ channels, and calcineurin. NMDA receptor stimulation also increases nuclear location and interaction with PYK2 of methyl-CpG binding domain protein 2 (MBD2), a modulator of histone modifications and nucleosome remodeling. In PYK2-KO neurons, MBD2 nuclear translocation is diminished, acetylation of histone H4-Lys5 is decreased, and methylation of histone H3-Lys4 is increased. The transcriptome is modified in PYK2-KO hippocampus with a decreased expression of genes coding for excitatory synaptic proteins. In cultured neurons, the absence of PYK2 enhances the glutamate-induced downregulation of synaptic protein transcripts related to epilepsy pathophysiology. In wild-type mice, pilocarpine-induced status epilepticus increases PYK2 and MBD2 nuclear localization in hippocampal neurons, especially in CA3. In PYK2-KO mice, aberrant synaptic sprouting and cell death triggered by status epilepticus are reduced in CA3 compared to wild-type littermates. In PYK2-KO neurons in culture, glutamate-induced cell death is attenuated, and this effect is abolished by re-expression of wild-type PYK2 but not of mutated PYK2 unable to translocate to the nucleus. In summary, our study indicates that regulated PYK2 nuclear translocation in hippocampal neurons may facilitate transcription by removing MBD2 from the active chromatin and may contribute to seizure-induced neurotoxicity.

## Introduction

Proline-rich tyrosine kinase 2 (PYK2) is a Ca^2+^-activated non-receptor tyrosine kinase belonging to the focal adhesion kinase (FAK) family [1]. PYK2 is enriched in adult hippocampal neurons [2, 3] and plays a role in neuronal plasticity and hippocampus-related memory [4–7]. In neurons, PYK2 is mostly localized in perikarya and dendrites [3, 8], and most studies on PYK2 addressed its function in the cytoplasm and synapses [9]. PYK2 nuclear accumulation was reported in various cell types or following mutations [10–12]. In neurons or PC12 cells, membrane depolarization or presynaptic activation induces PYK2 nuclear accumulation through Ca^2+^-activation of calcineurin [13]. PYK2 undergoes continuous cytonuclear shuttling with nuclear import due to the presence of nuclear localization and nuclear targeting sequences (NLS and NTS, respectively), while nuclear export is mediated by a nuclear export sequence (NES) regulated by phosphorylation of Ser778, a substrate of calcineurin [14]. However, the role of PYK2 in the nucleus of neurons is not known.

In non-neuronal cells, nuclear FAK and PYK2 interact with tumor suppressor p53 and enhance its proteosomal degradation [15, 16]. PYK2 and FAK also bind to methyl-CpG-binding domain protein 2 (MBD2) through their C-terminal focal adhesion-targeting domain (FAT), which binds to the Gly/Arg-rich region and MBD domain of MBD2 [17]. MBD2 belongs to a family of MBD domain-containing proteins, which associate with methylated CpGs, including MBD1-4 and MeCP2, mutated in Rett syndrome [18]. MBD2 recruits chromatin-remodeling complexes and histone deacetylases to methylated DNA [19]. MBD2 mRNA is enriched in the hippocampus and increases following ischemia [20]. MBD2-KO mice display a mild phenotype, including a slight maternal behavior alteration [21, 22]. Interaction of FAK with MBD2 regulates chromatin organization and gene expression [17, 23]. Although both PYK2 and MBD2 are enriched in the hippocampus, their interaction is not known.

Here, we study PYK2 cytonuclear trafficking and association with MBD2 in hippocampal neurons in culture in response to glutamate stimulation and examine the consequences of PYK2 KO on histone modifications and gene expression. We then explore the potential role of PYK2 nuclear translocation in a mouse model of pilocarpine-induced status epilepticus and cell death.

## Results

### Glutamate-induced PYK2 nuclear translocation in hippocampal neurons through activation of NMDA receptors and calcineurin requires its nuclear localization/targeting sequences

**Fig. 1:**
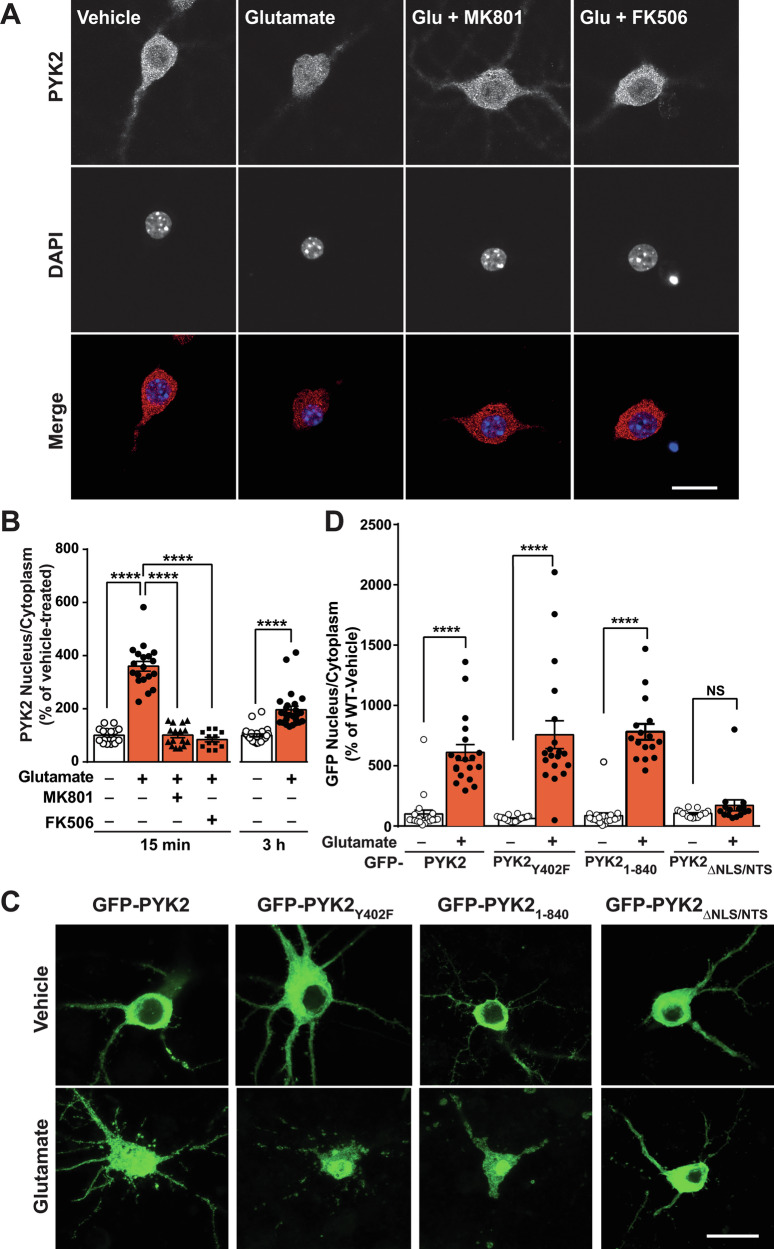
Glutamate induces PYK2 translocation into the nucleus. **A**, **B** Hippocampal neurons were cultured for 20–21 DIV and treated for 15 min with vehicle or glutamate (Glu, 40 µM) without or with MK801 (10 µM) or FK506 (1 µM), added 30 min before. A second set of cultured hippocampal neurons was treated for 15 min with vehicle or glutamate (Glu, 40 µM), placed in fresh medium and fixed 3 h later. Cultures were fixed and labeled for PYK2 immunoreactivity (**A**) to calculate the PYK2 IOD intensity ratio between the nucleus (identified with DAPI staining) and the cytosol (**B**). Data at 15 min were analyzed with Kruskal–Wallis’ test, *p* < 0.0001, *n* = 12–19 per group and at 3 h with Mann–Whitney’s test (*U* = 26, *p* < 0.0001, *n* = 24, 25 per group). **C** Hippocampal neurons cultured for 20–21 DIV were transfected with plasmids coding for GFP fused to wild-type PYK2, PYK2_Y402F_, PYK2_1–840_, or PYK2_ΔNLS/NLS_, as indicated, and treated with vehicle or glutamate (40 µM) for 15 min. Neurons were fixed 3 h after Glu treatment and imaged for GFP fluorescence. **D** Quantification of PYK2 nucleus/cytoplasm IOD intensity ratio. Kruskall–Wallis’ test, *p* < 0.0001, *n* = 15–24. **B**, **D** individual data points and means + SEM are shown, post-hoc multiple comparisons with Dunn’s test, ****p < 0.001. **A**, **C** images are single confocal optical sections, scale bar, 20 µm. Detailed statistical analyses in Supplementary Table 1.

In vehicle-treated 21-DIV hippocampal neurons in culture, endogenous PYK2 was preferentially localized in the soma and dendrite cytoplasm (Fig. 1A, B, all statistical analyses in Supplementary Table 1). Glutamate (40 µM, 15 min) induced PYK2 translocation into the nucleus, which was prevented by pretreatment with 10 µM MK801 or 1 µM FK506 (Fig. 1A, B), showing the involvement of NMDA receptors and calcineurin in the effects of glutamate. To examine the duration of the effect, the medium was replaced after a 15-min vehicle or glutamate application, and cells were fixed 3 h later for immunolabeling. PYK2 nucleus/cytoplasm immunoreactivity ratio in these glutamate-treated cultures was slightly higher than in cells treated with vehicle, but lower than at 15 min (Fig. 1B, right graph), indicating the reversibility of PYK2 nuclear accumulation.

To determine molecular properties of PYK2 important for its glutamate-induced nuclear accumulation in hippocampal neurons, we transfected wild-type (WT) or mutated GFP-PYK2 in cultured neurons, and treated them with vehicle or glutamate (Fig. 1C). Mutation of the autophosphorylation site (GFP-PYK2_Y402F_) or deletion of the C-terminal FAT domain and PR3 proline-rich motif (GFP-PYK2_1-840_) did not prevent nuclear translocation (Fig. 1C, D). In contrast, glutamate-induced nuclear accumulation was abolished in GFP-PYK2_ΔNLS/NTS_, in which key residues in the NLS (R_184_A/R_185_A) and NTS (S_747_A/T_749_A) were replaced by alanine (Fig. 1C, D). These results show that the nuclear translocation sequences previously identified in PC12 cells [14] control glutamate-induced PYK2 nuclear localization in hippocampal neurons.

### MBD2 associates with PYK2 in hippocampal neurons and translocates to the nucleus in response to glutamate

**Fig. 2:**
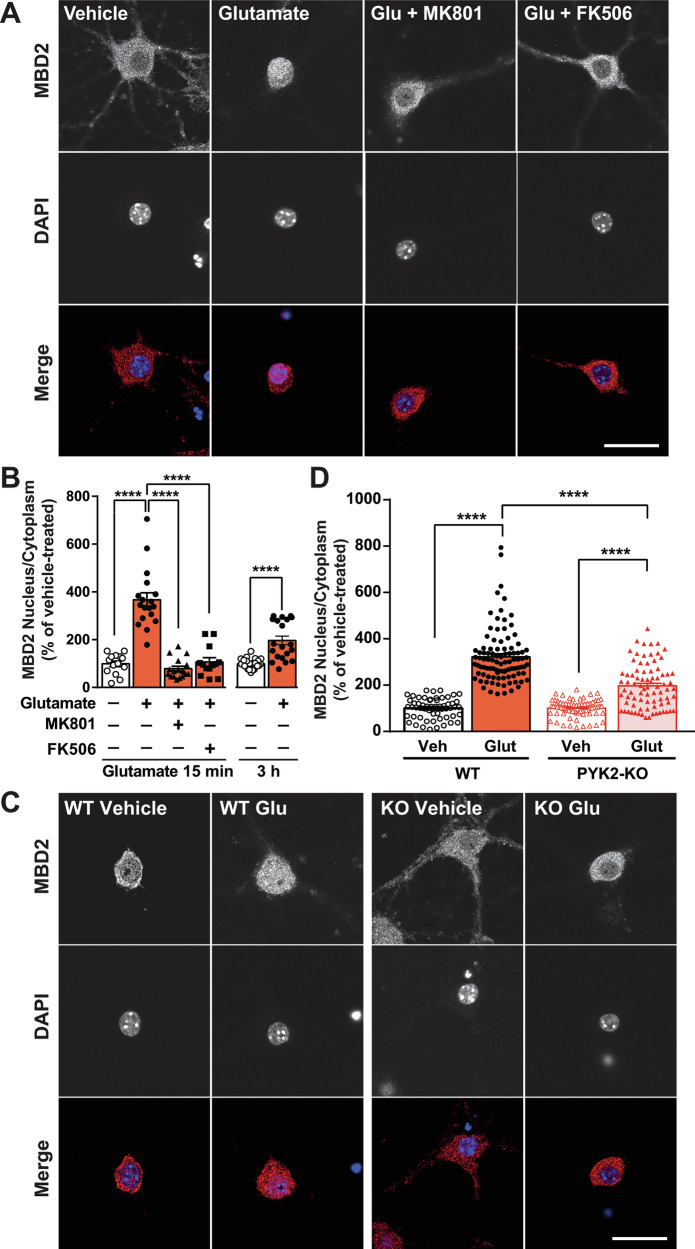
MBD2 translocates into the nucleus upon glutamate stimulation. **A** Hippocampal neurons (DIV20-21) were treated for 15 min with vehicle or glutamate (Glu, 40 µM) without or with MK801 (10 µM) or FK506 (1 µM), added 30 min before glutamate. Cells were fixed and labeled for MBD2 immunoreactivity. **B** Quantification of results as in (**A**) or from cells treated with glutamate (40 µM) for 15 min, 3 h before fixation. The nucleus/cytoplasm MBD2 immunofluorescence IOD ratio was quantified. For 15-min treatment, Kruskal–Wallis test, *p* < 0.0001, *n* = 13–18 per group, for 3-h treatment, two-tailed Mann–Whitney’s test, *U* = 26, *p* < 0.0001. **C** WT and PYK2-KO hippocampal neurons were treated for 15 min with vehicle (Veh) or glutamate (40 µM) for 15 min as in (**A**) and immunostained for MBD2. **D** Nucleus/cytoplasm MBD2 immunofluorescence intensity ratio. Kruskal–Wallis’ test, *p* < 0.0001, *n* = 57–97 per group. **B**, **D** individual data points and means + SEM are shown, post-hoc multiple comparisons with Dunn’s test, ****p < 0.0001. **A**, **C** scale bar, 20 µm. Detailed statistical analyses in Supplementary Table 1.

Because MBD2 is expressed in the hippocampus [24], where PYK2 is highly enriched [2, 3], we examined whether these proteins are associated in hippocampal neurons. We immunoprecipitated PYK2 from hippocampal neuronal cultures and immunoblotted pellets against MBD2. The two proteins co-immunoprecipitated (Supplementary Fig. 1). In contrast to other cell types where MBD2 is predominantly nuclear [17, 25], in vehicle-treated hippocampal neurons in culture, MBD2 immunoreactivity was high in the cytoplasm (Fig. 2A). Glutamate (40 µM, 15 min) increased nucleus/cytoplasm MBD2 immunofluorescence intensity ratio and this effect was less pronounced at 3 h (Fig. 2B). MBD2 nuclear accumulation was prevented by pretreatment with MK801 or FK506 (Fig. 2A, B). To determine whether PYK2 contributed to MBD2 localization, we used cultured hippocampal neurons from previously characterized PYK2-KO mice [2, 4], treated with vehicle or glutamate (Fig. 2C, Supplementary Fig. 2). In PYK2-KO cells, glutamate-induced MBD2 nuclear translocation was partly reduced (Fig. 2C, D). These results show that in hippocampal neurons, nuclear MBD2 is increased by glutamate treatment and that PYK2 contributes to this glutamate-induced nuclear accumulation.

### Glutamate-induced PYK2 and MBD2 nuclear translocation requires L-type Ca^2+^ channels and is accompanied by their molecular colocalization

**Fig. 3:**
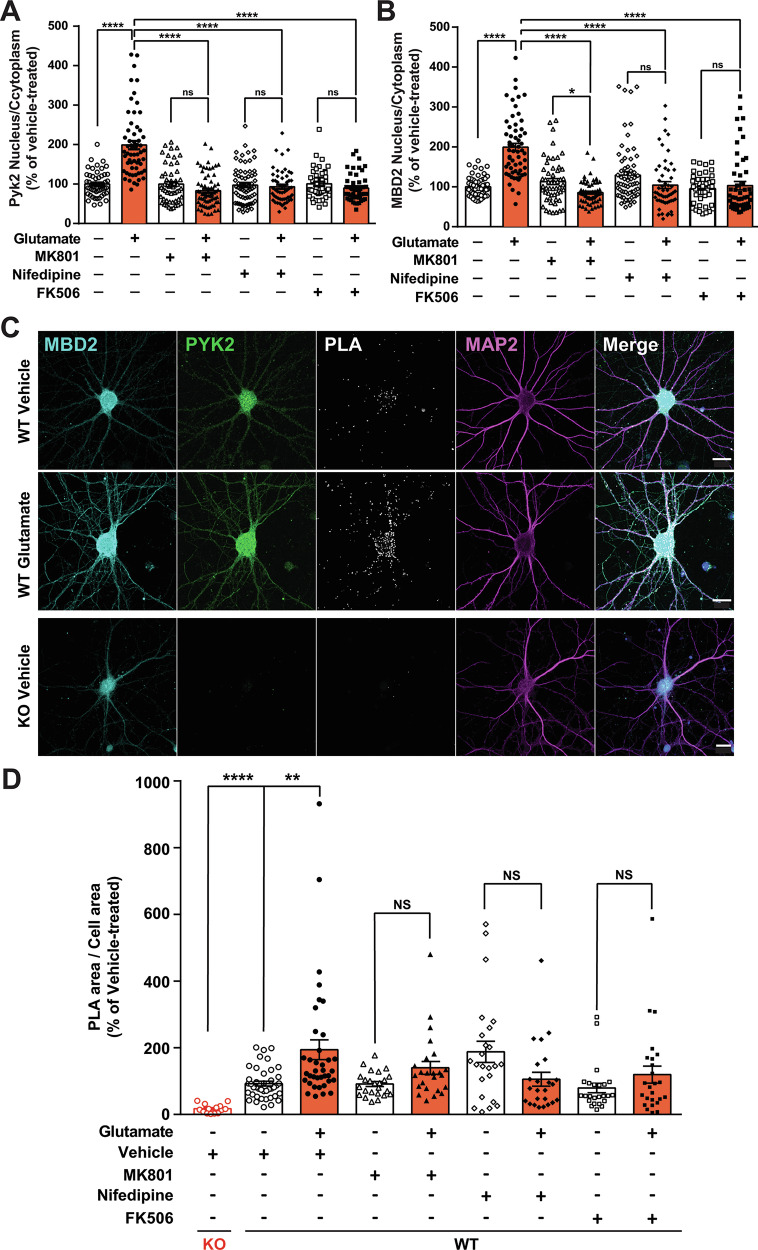
PYK2 and MBD2 nuclear translocation: role of Ca^2+^ and interaction revealed by PLA. Hippocampal neurons in culture were used for monitoring nuclear translocation of PYK2 and MBD2 and their interaction with PLA. **A**, **B** Quantification of PYK2 and MBD2 nuclear localization in response to glutamate 40 µM for 15 min and vehicle or various pharmacological inhibitors (MK801, 10 µM, nifedipine, 1 µM, or FK506, 1 µM) added 30 min prior to glutamate, was carried out in conditions similar to those used below for PLA assays (below). The immunoreactivity IOD ratio between the nucleus and the cytosol was calculated in each cell as in Fig. 1. **A** Translocation of PYK2, Kruskal–Wallis test, *p* < 0.0001, *n* = 46–68 per group. **B** Translocation of MBD2, Kruskal–Wallis test, *p* < 0.0001, *n* = 46–68 per group. **C** PLA was used to explore the interaction of PYK2 and MBD2 in cells. WT neurons were incubated with vehicle or glutamate, as in **A**, **B** (upper and middle row), and PYK2-KO neurons were incubated with vehicle as a control of the PLA specificity (lower row). Fixed neurons were labeled with PYK2, MBD2, and MAP2 antibodies. The PLA reaction was carried out with secondary antibodies labeled with complementary DNA strands and amplified DNA detected with Cy3-labeled oligonucleotides (PLA orange). Stacked confocal sections are shown. Scale bar 20 µm. **D** Quantification of results as in (**C**) using the ratio of the PLA area over the cell area. Kruskal–Wallis test, *p* < 0.0001, *n* = 24–41 per WT group, 14 for KO. **A**, **B**, **D** individual data points and means +/- SEM are shown, post-hoc multiple comparisons with Dunn’s test, NS, not significant, *p < 0.05, **p < 0.001, ****p < 0.0001. Detailed statistical analyses in Supplementary Table 1.

We then examined whether the Ca^2+^ influx through L-type Ca^2+^ channels, mostly located in cell bodies and proximal dendrites [26], contributed to PYK2 and MBD2 nuclear accumulation. We carried out experiments similar to those shown above, including, in addition, a pretreatment with 1 µM nifedipine, an L-type Ca^2+^ channel blocker (Fig. 3A, B). The glutamate-induced increase in PYK2 and MBD2 nucleus/cytoplasm ratio was prevented by nifedipine, like by MK801 and FK506 (Fig. 3A, B, Supplementary Fig. 2A, B). These results show that both NMDA receptors and L-type Ca^2+^ channels contribute to glutamate-induced nuclear translocation of PYK2 and MBD2, through Ca^2+^ influx and calcineurin activation.

Co-immunoprecipitation of PYK2 and MBD2 was not modified by glutamate treatment (Supplementary Fig. 1). We therefore investigated their in situ molecular interaction with proximity ligation assay (PLA, Fig. 3C). Immunofluorescence of the two proteins in untreated hippocampal neurons in culture was widespread (Fig. 3C stacked confocal images with PYK2/MBD2 in which lower nuclear concentration is not visible, but see single sections Supplementary Fig. 2A). In vehicle-treated cells, some PLA signal was detected over cell bodies and dendrites identified by MAP2 immunoreactivity (Fig. 3C, upper row). This PLA signal was specific because it was not visible in PYK2-KO neurons (Fig. 3C, lower row, Fig. 3D). In WT neurons, glutamate treatment increased the PLA signal over cell bodies, including nuclei, and dendrites (Fig. 3C, middle row, Fig. 3D). We did not observe any significant increase in PLA signal in response to glutamate when cells were pretreated with MK801, nifedipine or FK506 (Fig. 3D), indicating that the colocalization and molecular interaction of PYK2 and MBD2 is dependent on NMDA receptors, Ca^2+^ channels, and calcineurin. Taken together, our results show that, in hippocampal neurons, MBD2 is not exclusively nuclear and interacts with PYK2 in the cytoplasm and nucleus, and that this interaction is increased by glutamate treatment.

### Glutamate increases PYK2 and MBD2 in nucleoplasm and nucleoli, and MBD2 nuclear localization is altered in the absence of PYK2

**Fig. 4:**
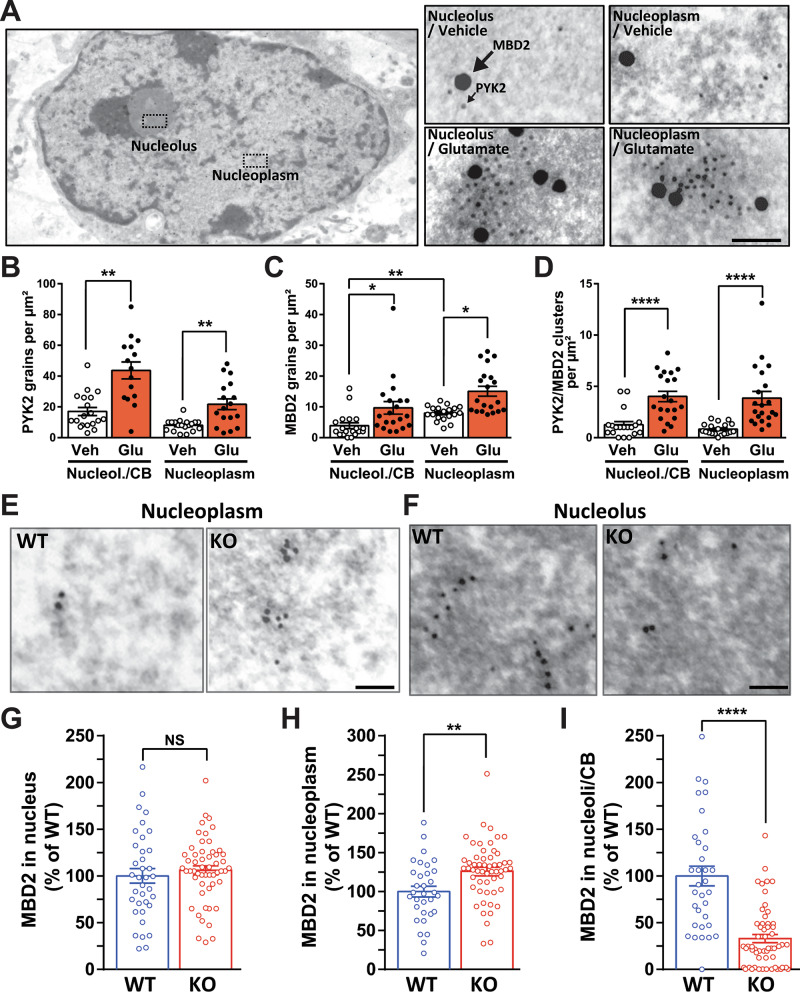
PYK2 and MBD2 intranuclear distribution and interaction investigated with electron microscopy. **A**–**D** WT hippocampal neurons were cultured for 20–21 DIV and treated for 15 min with vehicle or glutamate (Glu, 40 µM). Fifteen minutes after treatment with glutamate, cultures were processed for electron microscopy (see Material and Methods). **A** Transmission electron microscopy picture. Left picture, low magnification showing an example of nuclear areas used for quantification in nucleoli and nucleoplasm. Right: higher magnification pictures showing double immunogold labeling for PYK2 (10-nm particles) and MBD2 (20-nm particles). Scale bars, 1.5 µm left picture, 75 nm, right pictures. **B** Quantification of PYK2-associated gold particles in the nucleoplasm and nucleolus/coiled bodies (CBs) in vehicle (Veh) or glutamate (Glu)-treated cells, Kruskal–Wallis test, *p* < 0.0001. **C** Quantification of MBD2 particles, Kruskal–Wallis test, *p* < 0.0001. **D** Quantification of PYK2/MBD2 gold particle clusters, Kruskal–Wallis test, *p* < 0.0001. **B**–**D** *n* = 15–21 nuclei per group, data are individual data points and means +/- SEM. **E**, **F** Dorsal hippocampus CA3 tissue from adult WT and PYK2-KO mice was immunogold-labeled for MBD2 (10-nm particles). Representative images from WT and PYK2-KO mice are shown for nucleoplasm (**E**) and nucleolus/CBs (**F**). Scale bars, 40 nm. **G** Quantification of the MBD2 particles density (number of particles per µm²) in the whole nucleus, expressed as a % of the WT mean in the same experiments (three mice in each group, 2 slices per mouse). Mann–Whitney’s test, *p* = 0.29, *n* = WT, 36, KO, 54. **H** Quantification of MBD2 particles in nucleoplasm. Mann–Whitney’s test, *p* = 0.0017, *n* = WT, 32, KO, 55. **I** Quantification of MBD2 particles in nucleosomes/CBs. Mann-Whitney’s test, *p* < 0.0001, *n* = WT, 33, KO, 55.

We performed electron microscopy using immunogold double-labeling of PYK2 and MBD2 in hippocampal neurons in culture. In vehicle-treated neuronal nuclei, MBD2 and PYK2 immunogold particles were detected in nucleoplasm and nucleoli (Fig. 4A). PYK2-like immunoreactive particles were slightly but not significantly enriched in nucleoli/coiled bodies (CB, Fig. 4B), whereas MBD2-like immunoreactive particles were preferentially found in nucleoplasm (Fig. 4C). Glutamate treatment increased the number of gold particles corresponding to PYK2 (Fig. 4B) and to MBD2 (Fig. 4C) in both nucleoli/CB and nucleoplasm. To estimate the possible association between the two proteins, we counted the PYK2/MBD2 clusters (inter-immunogold particles distance < 200 nm) and found that cluster number was increased by glutamate treatment in nucleoplasm and nucleoli/CB (Fig. 4D). These ultrastructural results showing an increase in nuclear PYK2 and MBD2 and their clustering following glutamate treatment confirmed those obtained with immunofluorescence and PLA. They also showed the presence of the two proteins in both nucleoplasm and nucleoli/CB.

We then investigated the effects of PYK2-KO on the intranuclear distribution of MBD2-targeted immunogold particles in hippocampal pyramidal neurons from adult mice (Fig. 4E–I). The number of MBD2-positive particles in the entire nucleus was not changed in PYK2-KO compared to WT hippocampus (Fig. 4G). However, we observed an increase in the number of MBD2-immunogold particles in the nucleoplasm (Fig. 4E, H) and a decrease in the nucleoli/CB (Fig. 4F, I). These results suggested that in the absence of PYK2, MBD2 shifted from nucleoli/CB to the nucleoplasm, where euchromatin is located. Euchromatin is the major site of mRNA transcription, whereas the nucleolus and CB are respectively implicated in the processing of rRNA and snRNA [27]. Because MBD2 is a component of the nucleosome remodeling and deacetylase (NuRD) complex and can interact with other chromatin modifiers [19, 28], these observations raised the possibility of changes in chromatin modifications and/or transcription in PYK2-KO mice.

### Histone modifications are altered in the hippocampus of PYK2 KO mice

**Fig. 5:**
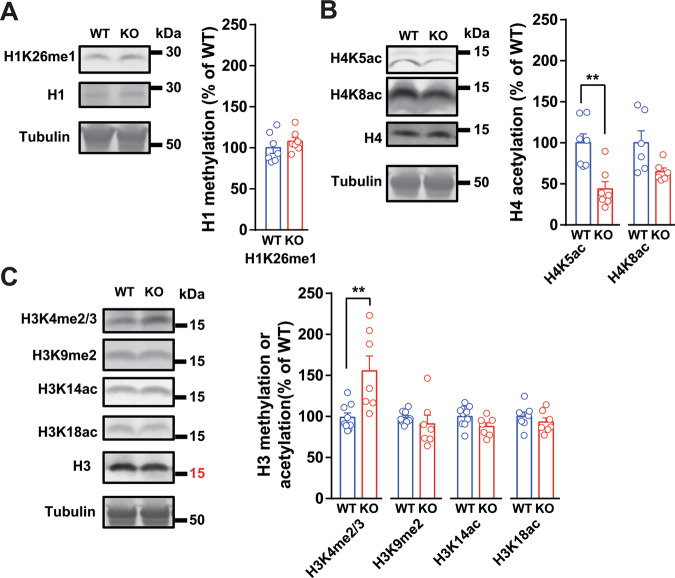
Selective alterations of histone modifications in the hippocampus of PYK2-KO mice. **A** Immunoblotting analysis of H1 monomethylated on lysine26 (H1K26me1) in hippocampal homogenates from WT and PYK2-KO mice. Tubulin was used as a loading control. The graph on the right shows the quantification of results, normalized to tubulin in the same samples and expressed as a % of the mean in WT. Statistical analysis two-tailed Mann–Whitney’s test *p* = 0.38, *n* = 7 in each group. **B** Immunoblot analyses for histone H4 acetylation on lysine 5 (H4K5ac) and lysine 8 (H4K8ac). Two-tailed Mann–Whitney’s test, H4K5ac, *p* = 0.004, *n* = 7 in each group, H4K8ac, *p* = 0.06, *n* = 6 in each group. **C** Immunoblot analyses for histone H3 di/trimethylation on lysine 4 (H3K4me2/3), monomethylation on lysine 9 (H3K9me), acetylation on lysine 14 (H3K14ac) and lysine 18 (H3K18ac). Two-tailed Mann–Whitney’s test, H3K4me2/3, 0.003, H3K9me, 0.11, H3K14ac, 0.07, H3K18ac, *p* = 0.54, *n* = 9 in each WT group except H3K18ac, *n* = 7 and *n* = 7 in all KO groups. Individual data points are shown with means + SEM, **p < 0.001. Detailed statistical analyses are in Supplementary Table 1 and see full-length blots in the original data of Fig. 5.

Because the absence of PYK2 has the potential to alter histone post-translational modifications through various mechanisms, including the change in MBD2 nuclear localization, we investigated several histone marks by immunoblotting hippocampal extracts from WT and PYK2-KO mice (Fig. 5A–C). H4K5ac, a transcriptional primer [29], was decreased in KO samples (Fig. 5B), compatible with an increased recruitment of NuRD/HDAC components to the chromatin as a consequence of the increased presence of MBD2 in this compartment in PYK2-KO mice. We also observed an increase in di/tri-methylated H3K4, an active mark [30], in KO mice (Fig. 5C). These alterations in chromatin modifications raised the possibility of an influence of PYK2 on transcription.

### PYK2-KO alters the transcriptome of hippocampal neurons with consequences on excitatory synapses

**Fig. 6:**
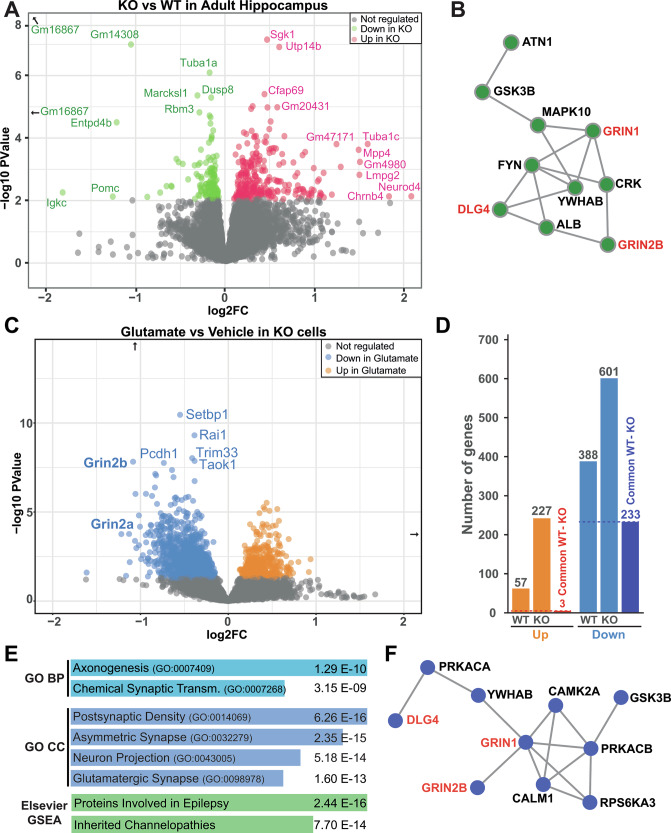
Transcriptomic effects of PYK2-KO in adult hippocampus and on glutamate effects in hippocampal cell culture. **A** Volcano plot of gene expression analyzed by RNAseq in the hippocampus of 4–5-month-old male WT and PYK2-KO mice (*n* = 4 per genotype), compared with DESeq2. Colors indicate significantly altered genes with a *P* value < 0.01 (off-scale genes are indicated by arrows, see detailed results in Supplementary Tables 2). **B** EnrichR network analysis of protein-protein interaction hub proteins identified from downregulated genes in PYK2-KO hippocampus (Supplementary Table 3b). NMDA receptor subunits and associated DLG4/PSD-95 are in red. **C** Transcriptomic effects of glutamate treatment (40 µM, for 2 h) in PYK2-KO hippocampal neurons in culture at DIV21 (*n* = 4 in each group, DSeq2, *P* value < 0.01, Supplementary Tables 5). **D** Comparison of the number of genes upregulated (Up) or downregulated (Down) by glutamate in WT and PYK2-KO cells in (**C**, **D**). The third column in each group indicates the number of common genes between WT and KO. **E** Top pathways: GO gene ontologies, BP biological process, CC cellular components, and Elsevier GSEA) identified from genes downregulated by glutamate in KO cells in (**D**, Supplementary Table 6b). **F** EnrichR network analysis of protein-protein interaction hub proteins identified from genes downregulated by glutamate in KO cells in (**D**) (Supplementary Table 6d). NMDA receptor subunits and associated DLG4/PSD-95 are in red.

We compared the transcriptome in the hippocampus of 2–3 month-old male WT and PYK2-KO mice using RNAseq. Many transcripts were altered in either direction (Supplementary Tables 2–3, Fig. 6A), indicating the contribution of PYK2 in gene expression. Gene ontology pathways (GO) of downregulated genes were related to synaptic function, especially excitatory/glutamatergic synapses (Supplementary Tables 3a, b). The hub protein networks deduced from protein-protein interaction of altered genes included glutamate NMDA receptors subunits (GRIN1, GRIN2B) and the associated protein PSD-95 (DLG4, Fig. 6B).

Comparison of transcriptomes of WT and PYK2-KO cultured hippocampal neurons, also revealed changes in the expression of many mRNAs (Supplementary Tables 4). They were different from those altered in adult hippocampus with few exceptions, including strong downregulation by Pyk2-KO, in both models, of transcripts from three genes (*Entpd4b*, *Gm16867*, *Gm27177*) located near *Ptk2b* gene locus on chromosome 14 and possibly subject to chromatin cis-interactions. Because of glutamate effects on PYK2 nuclear concentration and interaction with MBD2, we then asked if the absence of PYK2 modified the transcriptomic response of cultured hippocampal cells to glutamate. Glutamate (40 µM, 2 h) altered transcripts with a predominant downregulation more pronounced in KO than in WT cells (Supplementary Tables 5, Fig. 6C, D). The GO pathways of genes downregulated by glutamate in KO were related to excitatory glutamatergic synapses (Supplementary Tables 6, Fig. 6E). The deduced protein-protein interaction hub proteins network included GRIN1, GRIN2B and DLG4 (Supplementary Tables 6, Fig. 6F) and the top Elsevier GSEA pathway was related to epilepsy (Supplementary Tables 6, Fig. 6F). All these results supported a contribution of PYK2 in gene regulation, including genes related to synaptic function, which are downregulated in the absence of PYK2 in the hippocampus and in glutamate-treated cultured neurons. Because these genes scored high in relation to epilepsy pathophysiology, we tested this hypothesis in a mouse model of epilepsy.

### Pilocarpine-induced status epilepticus triggers nuclear translocation of PYK2 in pyramidal hippocampal neurons, predominating in CA3

**Fig. 7:**
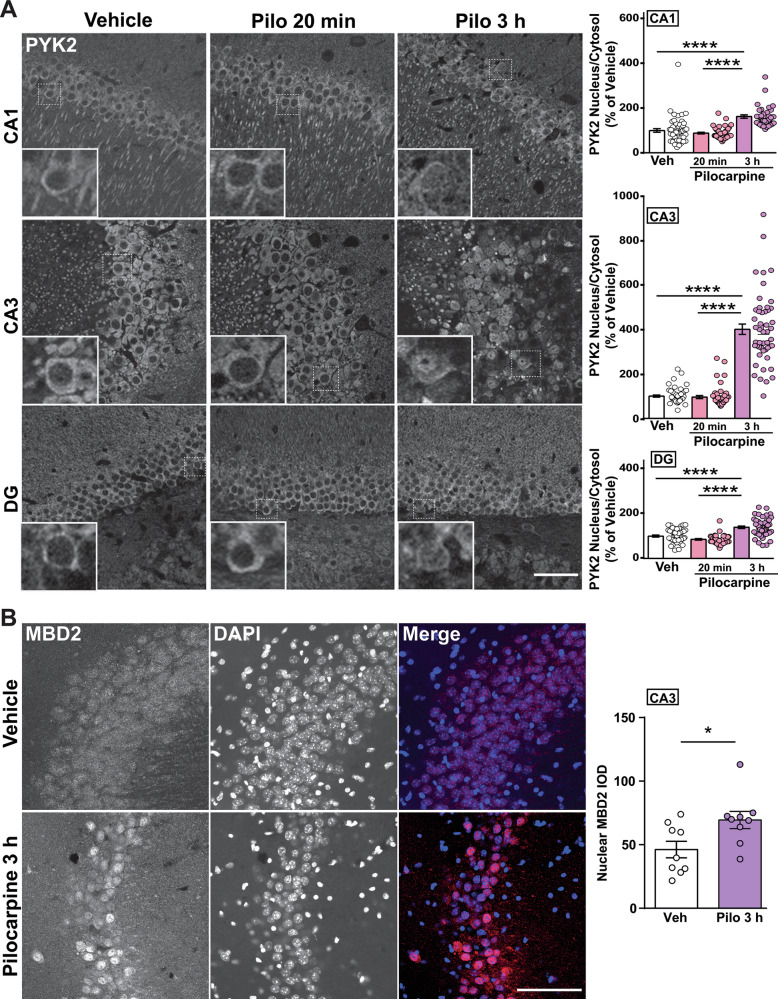
PYK2 and MBD2 translocation into the nucleus of hippocampal neurons upon pilocarpine treatment. **A** Adult WT mice were treated with lithium/pilocarpine (see Materials and Methods) and killed for histological examination 20 min or 3 h after *status epilepticus* (SE) onset and compared with vehicle-treated mice. Left side: confocal microscopy images of CA1, CA3 and DG immunostained for PYK2. Insets show higher magnification of the area indicated by a dashed rectangle. Scale bar, 40 µm, inset 10 µm. Right side: the PYK2 immunofluorescence IOD intensity ratio between the nucleus and cytoplasm was calculated (nuclei contours were determined by DAPI staining, not shown). Quantification was carried out in three mice per group and two sections per mouse. In each region, the data were analyzed with Kruskall–Wallis’ test: CA1, *p* < 0.001, *n* = 37–52 neurons per group, CA3, *p* < 0.001, *n* = 47–50, DG, *p* < 0.001, *n* = 43–49. Group comparison was done with Dunn’s test, ***p < 0.001. **B** Mice were treated with vehicle or lithium/pilocarpine as in (**A**). Pilocarpine-treated mice were sacrificed 3 h after SE onset and control mice at a similar time after vehicle treatment. Left side: immunostaining for MBD2 and DAPI nuclear staining in CA3 region, 3 h after SE onset. Scale bar, 100 µm. Right side: the nuclear IOD of MBD2 immunofluorescence was quantified. Student’s *t* test, *t*_16_ = 2.49, *p* = 0.024, *n* = 9 in each group. See Supplementary Table 1 for detailed statistical analyses.

We explored PYK2 nuclear translocation in a mouse model of epilepsy, pilocarpine-induced *status epilepticus* (SE) [31, 32]. This condition induces a synaptic release of glutamate [33] and activates PYK2 and tyrosine phosphorylation pathways at the post-synaptic level [34, 35]. We collected brain samples 20 min after vehicle treatment and 20 min or 3 h after SE onset (mice that did not develop a clear SE were excluded). In hippocampal neurons from vehicle-treated WT mice, PYK2 was mostly localized in the soma and apical dendrites (Fig. 7A). In pilocarpine-injected mice, PYK2 immunoreactivity in the nucleus was increased in CA1, CA3, and DG, 3 h after SE onset (Fig. 7A). The increase in nuclear PYK2 was more pronounced in CA3 than in CA1 and DG. This experiment showed that pilocarpine-induced SE resulted in a delayed nuclear translocation of PYK2 in neurons from all hippocampal regions, which was more pronounced in CA3. We then studied nuclear MBD2 immunofluorescence in CA3 and found it increased 3 h after SE, compared to vehicle-treated mice (Fig. 7B). We also observed an increase in phosphorylation of histone H3 on Ser9 and nuclear immunofluorescence of FOS and EGR1, two immediate-early gene products, which are transcription factors (Supplementary Fig. 3). These observations supported the effects of SE on chromatin regulation [32, 36, 37] in CA3, known to be highly susceptible to neuronal loss and spreading of epileptic activity in temporal lobe epilepsy models [38–40].

### In PYK2-KO mice, SE-induced delayed behavioral phenotype is slightly reduced

**Fig. 8:**
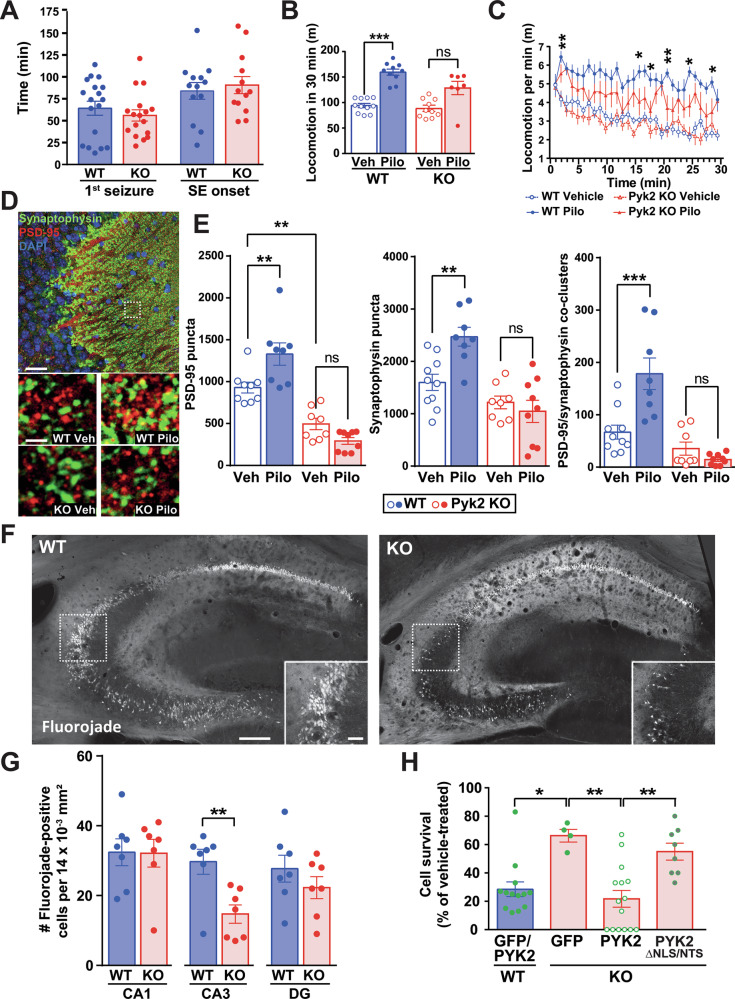
PYK2-KO decreases the neurotoxic effects of pilocarpine-induced epilepsy in vivo and glutamate excitotoxicity in vitro. **A** WT and PYK2-KO adult mice were subjected to lithium/pilocarpine treatment (see Materials and Methods). The mice were visually monitored, and the time to reach the first seizure and the SE onset were recorded. Time of first seizure, Mann–Whitney’s test, *p* = 0.94, *n* = 17, 18 mice per group, time of SE, two-tailed *t* test, *p* = 0.64, *n* = 13 per group. **B**, **C** Locomotor activity of WT and PYK2-KO in an open field, 7 days after treatment with vehicle or lithium/pilocarpine (only mice that had displayed a SE were included). **B** Total locomotion during 30 min, Kruskal–Wallis’ test, *p* < 0.0001, group comparison with Dunn’s test, ***p < 0.001. **C** The same data are shown as a time-course in 1-min bins. Two-way repeated measure ANOVA, interaction, F_(87, 986)_ = 1.24, *p* = 0.07, time effect, F_(29, 986)_ = 8.13, *p* < 0.0001, group effect, F_(3, 34)_ = 24.96, *p* < 0.0001. For each time point group comparisons with Holm-Sidak’s multiple comparisons test (see Supplementary Table 1), significant differences between pilocarpine-treated WT and PYK2-KO mice are indicated, *p < 0.05, **p < 0.01. **D** The day after open field (8 days after vehicle or pilocarpine treatment), WT and PYK2-KO mice were perfused, and tissue was used for immunohistofluorescence. Effects of pilocarpine-induced SE on synaptophysin and PSD-95 immunostaining in CA3 (top, low magnification in WT vehicle-treated, scale bar 50 µm, bottom, higher magnification of areas located as indicated by the dashed rectangle, scale bar, 2 µm). **E** The number of PSD-95-positive, synaptophysin-positive puncta, and their colocalized clusters was quantified in vehicle-treated and pilocarpine-treated WT and PYK2-KO mice, sacrificed 8 days after SE. One-way ANOVA for all p < 0.0001. Post-hoc Dunn’s test, **p < 0.01, ***p < 0.001, ns not significant. **F** Tissue as in (**D**), fluoro-Jade staining. Scale bar, 50 µm, inset, 10 µm. **G** Fluoro-Jade-positive cells were counted in each hippocampal region and compared between WT and PYK2-KO mice with Mann–Whitney’s test: CA1, *p* = 0.78, CA3, *p* = 0.007, DG *p* = 0.39, *n* = 7 mice per group. **H** Cultured hippocampal neurons from WT and PYK2-KO embryos were transfected at DIV 18-19 with plasmids coding for GFP or GFP fused to wild-type PYK2 or PYK2_ΔNLS/NST_ (i.e., with mutations of nuclear addressing sequences). Two days later, neurons were treated with vehicle or glutamate (125 µM), fixed after 24 h, and imaged for GFP fluorescence. The number of GFP-positive neurons per slide was counted in vehicle and glutamate-treated cultures and expressed as a percentage of the number of GFP-positive neurons in control cultures treated with vehicle (not shown). The percentage of surviving GFP-positive neurons was analyzed with Kruskal–Wallis’ test, *p* < 0.0001 and groups compared with Dunn’s test, *p < 0.05, **p < 0.01, *n* = 4–15. See Supplementary Table 1 for detailed statistical analyses.

We next asked if PYK2 played a role in behavioral consequences of pilocarpine-induced epilepsy. Adult WT and PYK2-KO mice subjected to pilocarpine-induced SE, displayed no difference between genotypes in the time of first seizure and SE onset (Fig. 8A). To test if PYK2 deletion modified delayed consequences of pilocarpine-induced SE, we evaluated spontaneous locomotor activity in the open field, which increases several days after pilocarpine administration [41]. One week after pilocarpine treatment, the increase in total locomotion was blunted in KO mice (Fig. 8B) and time-course analysis showed that pilocarpine-induced increase in locomotion was less pronounced in PYK2-KO than in WT mice (Fig. 8C). In pilocarpine-treated mice, the time spent in center was decreased in WT, reflecting an anxious-like behavior, but proportionally less in KO (Supplementary Fig. 4A). The parallel index, which evaluates mice gait and is altered in epileptic mice [42], was increased in pilocarpine-treated WT mice, after habituation to the apparatus, but not in KO (Supplementary Fig. 4B, C). These results indicated that the absence of PYK2 did not change the effects of pilocarpine on seizures, but decreased some long-term behavioral consequences.

### PYK2-KO prevents SE-induced aberrant synaptic sprouting in CA3

Because pilocarpine-induced SE causes pathological synaptic plasticity, including the formation of new spines and axonal sprouting [43], we investigated synaptic markers, 8 days after SE onset in WT and PYK2-KO mice, in CA3 (Fig. 8D, E), where nuclear import of PYK2 was most altered. The number of PSD-95-positive puncta, a post-synaptic component of excitatory synapses, was lower in PYK2-KO than in WT mice, as previously reported [4]. In WT mice, pilocarpine treatment increased the number of puncta immunopositive for PSD-95 or synaptophysin, a presynaptic vesicle protein, and their colocalization clusters, indicative of active synapses (Fig. 8A, B). These changes were not observed in PYK2-KO mice, providing evidence that the absence of PYK2 prevented SE-induced aberrant synaptic sprouting.

### SE-induced hippocampal neuronal death in CA3 is reduced in PYK2-KO mice, and PYK2 nuclear translocation contributes to glutamate-induced neurotoxicity

Pilocarpine-induced status epilepticus is accompanied by histopathological alterations in the hippocampus, including neuronal degeneration and death [44]. We evaluated cell death by Fluoro-Jade staining 8 days after pilocarpine injection, a time corresponding to the peak of cell death in the hippocampus [45]. The number of Fluoro-Jade-positive cells in CA3 was reduced in PYK2-KO mice compared with WT mice, whereas it was not changed in CA1 and DG (Fig. 8F, G). Because CA3 was the region where pilocarpine-induced nuclear translocation of PYK2 was maximum, this result suggested that PYK2 nuclear translocation could participate in the cytotoxic effects of pilocarpine.

To assess the putative causal contribution of PYK2 nuclear translocation in cell death associated with pilocarpine-induced SE, we used a model of glutamate-induced neurotoxicity. We transfected WT and PYK2 KO hippocampal cells in culture at DIV 18-19 with GFP or PYK2 fused to GFP (GFP-PYK2). To test the role of nuclear PYK2 we also used GFP-PYK2_ΔNLS/NTS_, which cannot translocate to the nucleus [14] including in hippocampal neurons (see Fig. 1C, D). At DIV 20-21, hippocampal cell cultures were treated with vehicle or an excitotoxic concentration of glutamate (125 µM) and fixed 24 h later, to count the number of remaining GFP-positive cells. As compared to vehicle-treated neurons, glutamate induced a massive neuronal loss in WT neurons transfected with either GFP (% survival 27.8 ± 4.4, mean ± SEM, *n* = 6) or GFP-PYK2 (% survival 29.0 ± 9.2, *n* = 7). Glutamate-induced cell death was attenuated in PYK2-KO hippocampal cultures transfected with GFP (Fig. 8H). Transfection of PYK2-KO neurons with GFP-PYK2 restored glutamate-induced cell death to levels similar to WT cells (Fig. 8H), confirming that PYK2 contributes to glutamate-induced toxicity in these conditions. In contrast, transfection with GFP-PYK2_ΔNLS/ΔNTS_ did not restore the increased toxicity (Fig. 8H), providing evidence that nuclear translocation of PYK2 is important for its role in neurotoxicity. These results in a model of excitotoxicity in culture support the contribution of nuclear PYK2 to pilocarpine-induced neurotoxicity in vivo.

## Discussion

Here, we provide insights into the mechanisms and role of regulated PYK2 nuclear translocation in hippocampal neurons and its implication in a model of epilepsy. We show that PYK2 accumulates in the nucleus of hippocampal neurons upon glutamate stimulation through a mechanism that involves NMDA receptors and L-type Ca^2+^ channels, supporting a role of Ca^2+^ influx in dendrites and cell bodies. We show that the glutamate-induced increase in nuclear PYK2 is prevented by mutation of its nuclear localization [15] and targeting [14] sequences. In hippocampal neurons, nuclear translocation of PYK2 requires calcineurin activity, as in PC12 cells [13, 14], in which dephosphorylation of Ser778 by calcineurin unmasks the activity of PYK2 nuclear export sequence (residues 730–739) [14]. Thus, glutamate stimulation induces a change in the intraneuronal localization of PYK2, which switches from a diffuse somatodendritic distribution to an enrichment in both spines [4, 46] and the nucleus.

We show that, in hippocampal neurons, PYK2 interacts with the chromatin regulator MBD2. Glutamate increases the nuclear localization of both proteins and their clusterization. In PYK2-KO neurons, glutamate-induced MBD2 nuclear accumulation is decreased and its intranuclear distribution modified, shifting from nucleoli to nucleoplasm, where active chromatin is located. MBD2 participates in NuRD complexes, which deacetylate histones and interact with other chromatin modifiers [19, 28], remodeling nucleosomes and favoring a repressive chromatin conformation [47]. In PYK2-KO mice, we found a decrease in H4K5 acetylation, a widespread mark involved, for example, in priming genes associated with fear memory in the mouse hippocampus [29]. This decrease is consistent with the relocation of MBD2 to the chromatin observed in the absence of PYK2. Thus, by interacting with MBD2 and relocating it away from active chromatin, nuclear PYK2 may favor gene expression in response to Ca^2+^ influx [23].

In support of a role of PYK2 on transcription, our RNAseq results show that PYK2-KO alters gene expression in adult hippocampus and hippocampal neurons in culture. We observed both increased and decreased gene expression, consistent with PYK2 playing an indirect modulatory role, dependent on cell state, through the various signaling pathways it is involved in [48, 49]. Gene downregulation in PYK2-KO is also compatible with a loss of control exerted by PYK2 on MBD2, resulting in a repressive chromatin state. In the adult hippocampus, downregulated genes were related to excitatory synaptic function. Because PYK2 is involved in synaptic plasticity [6, 46], which is altered in the absence of PYK2 [4, 5], our current results support a dual contribution of PYK2 in the regulation of synapse properties and plasticity, directly at the post-synaptic level and indirectly, through modulation of synaptic-related gene transcription.

The multiple levels at which PYK2 can control excitatory synaptic properties raise the question of its role in epilepsy, which we explored in pilocarpine-induced SE. In this model, a long-lasting increase in the tyrosine phosphorylation of NMDA receptor subunits NR2A and NR2B and increased activity of PYK2 and Src in the post-synaptic region was reported [35]. Here, we show that SE increases PYK2 nuclear immunoreactivity in the principal neurons of all hippocampal regions, concomitant with MBD2 nuclear translocation. Although we don’t know the reason for the predominance of the effect in CA3, our results support a role of PYK2 in the consequences of pilocarpine-induced SE in this region, which is important in temporal lobe epilepsy models [38–40]. SE-induced aberrant synaptic sprouting was blocked in PYK2-KO mice, possibly accounting for the blunting of delayed behavioral alterations in these mice. In addition, as expected [50], SE-induced cell death in WT mice hippocampus, and in PYK2-KO mice, this effect was markedly decreased in CA3, where PYK2 nuclear translocation predominates in WT mice. PYK2 can participate in SE neurotoxic effects at various levels, given its roles at excitatory synapses [9]. Yet, our current results in a glutamate-excitotoxicity model in culture support a specific contribution of nuclear PYK2 because the partial protection of PYK2-KO cells against glutamate-induced neurotoxicity, was removed by transfection of WT-PYK2 but not of a mutant unable to translocate to the nucleus. In non-neuronal cells, nuclear PYK2 facilitates cell growth and survival by interacting with the tumor suppressor p53 [15] and contributes to tumor progression [48]. In contrast, PYK2 proapoptotic effects have also been reported [51], and PYK2 may contribute to neuronal death in a mouse model of ischemic stroke [52], but the role of nuclear PYK2 was not explored. Our findings open a new avenue to explore the role of PYK2 and its nuclear translocation in neurotoxicity in epilepsy and other conditions. Further work is needed to characterize the mechanisms by which nuclear PYK2 contributes to chromatin regulation and excitotoxic cell death. Although nuclear translocation does not require PYK2 activity and autophosphorylation [13, 14], it will be important to determine whether its kinase activity is implicated in toxic effects and whether it could be counteracted by kinase inhibitors, with potential therapeutic applications in epilepsy and ischemia.

## Materials and methods

### Animals

We used C57Bl/6 PYK2 KO mice generated by our laboratory in collaboration with Gen-O-way (Lyon, France), first by flanking with LoxP sequences, through homologous recombination, exons 15b to 18 of the Ptk2b gene which codes for PYK2 (PYK2^f/f^), and then crossing these mice with a “deleter” line expressing Cre in all cell types, generating a Ptk2b^−/−^ line with a constitutive deletion of PYK2, referred to as PYK2-KO [2]. These procedures and the mouse lines were extensively characterized [2, 4]. The deletion disrupts the protein kinase domain and prevents protein expression [4]. Mouse genotyping was done from a tail biopsy as described [4], in the lab or by Charles River services. Breeding strategy used crossing of heterozygous mice to generate +/+ and −/− progeny. Male WT and KO littermates were used. In experiments with only C56Bl/6 wild-type mice, animals were purchased from Janvier Laboratories (Le Genest-Saint-Isle, France). All mice were housed with *ad libitum* access to food and water in a colony room kept at 19–22 °C and 40–60% humidity, under a 12/12 h light/dark cycle. Animals were used in accordance with the ethical guidelines (European Community Guidelines, and French Agriculture and Forestry Ministry guidelines for handling animals, decree 87849, license A75-05-22). All methods were performed in accordance with the relevant ethical guidelines and regulations and approved by the *Ministère de l’Education Nationale de l’Enseignement Supérieur de la Recherche et de lʼInnovation* (France, APAFIS#26765).

### Primary hippocampal neurons culture and pharmacological treatments

Hippocampal neurons were prepared from E17 C57Bl/6 mouse embryos (pregnant mice from Charles River, Saint Germain Nuelles, France) or from our PYK2 mice colony as previously described [4]. The neuronal cell suspension was seeded (70,000 cells per cm²) on coverslips precoated with poly-D-lysine (0.1 mg ml^−1^, Sigma) in 24-well plates or in 6-well plates without coverslips. Neurobasal medium (Gibco-Thermo Fisher, Villebon-sur-Yvette, France) containing 1 mL per 50 mL of B27 supplement (Gibco) and 0.5 mL per 50 mL of GlutaMAX (100×, Gibco) was used to grow the cells in serum-free medium conditions and maintained at 37 °C in 5% CO_2_. At DIV21-22 cells were treated with vehicle or MK801, 10 µM, or FK506, 1 µM, or nifedipine, 1 µM, for 30 min (all drugs from Merck-Millipore-Sigma-Aldrich, Saint Quentin Fallavier, France). The concentrations and duration of application of these drugs were based on our previous work on the application of glutamate 40 µM for 15 min on PYK2 phosphorylation and its blockade by MK801 in neurons in culture [4], and blockade of PYK2 nuclear translocation by FK506 [13]. Nifedipine was used at a low concentration, selectively acting on L-type calcium channels [53]. Then, cells were treated with vehicle or sodium glutamate (40 µM, Sigma) for 15 min and samples were collected for immunoblot and immunofluorescence analysis or the glutamate was washed out, and cells were further incubated for 3 h before being fixed. In another set of experiments to study excitotoxicity, hippocampal cell cultures were treated with 125 µM glutamate and fixed 24 h later.

### Cell transfection and constructs

PYK2^+/+^ and PYK2^−/−^ hippocampal neurons at DIV 18 were transfected using Transfectin (Bio-Rad, Hercules, CA, USA) following manufacturer’s instructions and left for 48–72 h. Cells were transfected with GFP (control) or the previously described constructs [14], in which GFP was fused to the N-terminus of PYK2: GFP-PYK2, GFP-PYK2_1–840_ (PYK2 deleted from the FAT domain and the third proline-rich motif), GFP-PYK2_Y402F_ (PYK2 with a point mutation of the autophosphorylated tyrosine-402), GFP-PYK2_ΔNLS/NTS_ (PYK2 with point mutations in nucleus import motifs).

### Brain tissue preparation and immunofluorescence procedures

Animals were deeply anesthetized with pentobarbital (60 mg kg^−1^) and intracardially perfused with a 40 g l^−1^ paraformaldehyde solution in 0.1 M sodium phosphate buffer, pH 7.2. Brains were removed and post-fixed overnight in the same solution, cryoprotected with 300 g l^−1^ sucrose in PBS with 0.2 g l^−1^ sodium azide and frozen in dry-ice-cooled isopentane.

Serial coronal sections (30 µm-thick) obtained with a cryostat were processed for immunohistochemistry as free floating. The sections were washed three times in 0.2 M sodium phosphate buffer, pH 7.5 with 150 mM NaCl (PBS), permeabilized 15 min by shaking at room temperature in PBS with 3 ml l^−1^ Triton X-100 and 30 ml l^−1^ normal goat serum (Pierce Biotechnology, Rockford, IL, USA). After three washes, brain slices were incubated overnight by shaking at 4 °C with the primary antibodies in PBS with 0.2 g l^−1^ sodium azide. Slices were then washed three times and incubated 2 h under shaking at room temperature with fluorescent secondary antibodies.

Cells in culture were fixed by incubation for 10 min with 40 g l^−1^ paraformaldehyde in PBS. Fixed cells were permeabilized in 1 ml l^−1^ Triton X-100 in PBS for 10 min and then blocked for 1 h in PBS with 10 g l^−1^ bovine serum albumin. Cells were then incubated with primary antibodies at 4°C overnight and after three washes with PBS, incubated with the corresponding fluorescent secondary antibodies. Cells were then rinsed twice with PBS and incubated with DAPI (Sigma, 1:10,000) for 10 min in PBS. After washing twice with PBS, the coverslips were mounted with Vectashield (Vector laboratories Burlingam, UK).

The primary antibodies used were rabbit polyclonal antibodies for PYK2, 1:500 (# 07M4755, Merck-Millipore-Sigma-Aldrich), acetyl-lysine-5-histone 4 (H4K5ac), 1:750 (#07-327, Merck-Millipore-Sigma-Aldrich), synaptophysin 1:500 (#101011, SynSyn), EGR1 1:1000 (#4154S, Cell Signaling), phospho-Ser9 H3 1:250 (#AB10543, Abcam, Cambridge, UK), PSD-95 1:250 (#3450S, Cell Signalling), and acetyl-lysine-8-histone 4 (H4K8ac), 1:350 (#ab61237-100, Abcam, Cambridge, UK) or goat polyclonal antibodies for MBD2 (1:350, #ab58241, #ab61237-100, Abcam) or mouse cFOS 1:150 (#SC-166940, Santa Cruz). Corresponding fluorescent secondary antibodies, coupled to Cy3 or Cy2 (1:200) were from Jackson ImmunoReseach (West Grove, PA, USA). No signal was detected in control samples incubated in the absence of the primary antibody.

### Confocal imaging and analysis

Hippocampal neurons in culture or dorsal hippocampus sections were imaged using a Leica Confocal SP5-II with a ×10 or ×40 numerical aperture lens with standard (1 Airy disc) pinhole. Frame averaging (2 frames per *z*-step) was held constant throughout the study. Confocal *z*-stacks were taken every 0.2 μm for culture experiments and every 3 μm for tissue slices, and at 512 × 512 pixel resolution. For cytonuclear localization of PYK2 and MBD2, the ratio of the integrated optical density (IOD) in the nucleus and in the cytosol was measured in the stack with the largest area of DAPI (Sigma) staining. For synaptic clusters imaging (Synaptophysin and PSD-95) we performed *z*-stacks taken every 2 μm and quantified positive clusters and their colocalization as previously described [42]. For nuclear staining analyses in vivo IOD of the different hippocampal subfields CA1, CA3 and DG was evaluated in the pyramidal and granular layers respectively. The freeware NIH ImageJ (Wayne Rasband, NIH) was used for all imaging quantifications.

### Electron microscopy

Mice were deeply anesthetized with pentobarbital (60 mg kg-1) and transcardially perfused with a solution containing 40 g l^−1^ paraformaldehyde and 1% g l^−1^ glutaraldehyde in 0.1 M phosphate buffer, pH 7.4 (PB). After perfusion, brains were removed from the skull, and immersed in the same fixative for 12 h at 4 °C. Tissue blocks containing the hippocampus were dissected, washed with PB and cut with a vibratome to obtain 1 mm^3^-blocks. Neuronal cultures, after 21 days in vitro and treated with 40 µM glutamate or vehicle were fixed for 30 min with the same fixative solution used for the tissues and washed with PB.

Samples from tissues or cell culture were post-fixed with 20 g l^−1^ osmium tetroxide in PB for 20 min. They were dehydrated in a series of ethanol and flat embedded in epoxy resin (EPON 812 Polysciences, Warrington, PA, USA). After polymerization, blocks from the CA1 region or blocks from cell cultures were cut at 70 nm thickness using an ultramicrotome (Ultracut E, Leica). EPON embedded sections were ultrathin-sectioned with a diamond knife, picked up on Formvar-coated 200 mesh nickel grids. For etching resin and removing osmium, sections were treated with saturated aqueous sodium periodate (NaIO_4)_. Sections were sequentially double-immunolabelled for PYK2 and MBD2 with the same antibodies used for immunofluorescence (see above), revealed as described [54], with protein A-coupled gold particles (10-nm diameter, for PYK2 and 20-nm for MBD2) from CMC Utrecht (Netherlands). After immunolabeling the sections were double stained with uranyl acetate and lead citrate prior to observation with a Philips (CM-100) electron microscope. Digital images were obtained with a CCD camera (Gatan Orius). Concentrations for each antibody were adjusted such that they resulted in low background labeling, as assessed over areas devoid of gold particles. Immunoreactions were carried out several times. To test method specificity of the immunostaining procedure, the primary antibody was omitted. Under these conditions, no selective labeling was observed.

For particle counting in mouse hippocampus and in cell cultures the number of individual 10-nm and 20-nm gold particles localized in morphometrically determined areas was visually counted and the labeling density was calculated as the number of gold particles per µm² expressed as a percentage [55]. For gold particles clustering analysis we used quadrat analysis [56] using circular grids of 200-nm diameter, placing the core of the grid over all the 20-nm gold particles identified in the image. Density of 10-nm and 20-nm gold particles was counted in this area and density expressed as a percentage. The gold particle distribution was evaluated as a cluster when the number of particles was greater than 1 [56].

### Immunoblot analysis

Animals were euthanatized by cervical dislocation. The hippocampus was dissected out, frozen using CO_2_ dry-ice pellets and stored at -80 °C until use. Briefly, the tissue was lysed by sonication in 250 μl of lysis buffer (PBS containing 10 ml l^−1^ Nonidet P-40, 1 mM phenylmethylsulphonyl fluoride, 10 mg l^−1^ aprotinin, 1 mg l^−1^ leupeptin, 2 mg l^−1^ sodium orthovanadate). After lysis, samples were centrifuged at 12,000 rpm for 20 min. Supernatant proteins (15 μg) from brain regions extracts were separated by SDS-PAGE and transferred to nitrocellulose membranes (GE Healthcare, Chalfont St. Giles, UK). Membranes were blocked in TBS-T (150 mM NaCl, 20 mM Tris–HCl, pH 7.5, 0.5 ml l^−1^ Tween-20) with 50 g l^−1^ non-fat dry milk and 50 g l^−1^ BSA. Immunoblots were probed with the following antibodies (all diluted 1:1000): rabbit polyclonal antibodies for histone H1 (#ab62884, Abcam), monomethyl-Lys26-histone H1 (H1K26me, #ABE446, Merck-Millipore-Sigma-Aldrich), histone H3 (#ab1791, Abcam), acetyl-Lys14-histone H3 (H3K14ac, #07-353 Merck-Millipore-Sigma-Aldrich), acetyl-Lys18-histone H3 (H3K18ac, #ab1191, Abcam), histone H4 (#ab61255, Abcam), acetyl-Lys5-histone H4 (H4K5ac, #07-327, Merck-Millipore-Sigma-Aldrich), acetyl-Lys8-histone H4 (H4K8ac, #ab61237-100, Abcam), PYK2 (#074M4755, Merck-Millipore-Sigma-Aldrich); mouse monoclonal di/tri methyl-Lys4-histone H3 (H3K4me2/3, #mAbcam6000, Abcam); rabbit monoclonal for dimethyl-Lys9-histone H3 (H3K9me2, #04-768, Millipore); goat polyclonal for MBD2 (#ab58241, Abcam). All blots were incubated with the primary antibody overnight at 4 °C by shaking in PBS with 0.2 g l^−1^ sodium azide. After several washes in TBS-T, blots were incubated with secondary anti-rabbit or anti-mouse IgG IRdye800CW-coupled or anti-mouse IgG IRdye700DXcoupled antibodies (1:2000, Rockland Immunochemicals, USA). Secondary antibody binding was detected by Odyssey infrared imaging apparatus (Li-Cor Inc., Lincoln, NE, USA). For loading control a mouse monoclonal antibody for α-tubulin was used (#083M4847V, 1:10000, Sigma) was used.

## Immunoprecipitation

Protein (300 μg) were incubated overnight at 4 °C on a rotary mixer with anti-PYK2 antibody (#074M4755, Merck-Millipore-Sigma-Aldrich, 1 μg μl^−1^) in a buffer containing 50 mM Tris–HCl, pH 8.0, 150 mM NaCl, 1% IGEPAL CA-630 (Sigma-Aldrich), 2 mM PMSF, 2.5 mM NaF, 1 mM NaVO_4_ and 1:1000 protease inhibitor cocktail (Sigma-Aldrich). The immune complexes were precipitated overnight at 4 °C with the addition of 5% protein A-Sepharose Cl-4B (Sigma-Aldrich). Beads were collected by centrifugation, washed sequentially with immunoprecipitation buffer, immunoprecipitation buffer-PBS (1:1) and PBS, and then placed twice at 100 °C for 10 min in SDS sample buffer. Immunocomplexes were resolved on SDS-PAGE, and immunoblot analyses were carried out as described above.

### Proximity ligation assay (PLA)

Duolink PLA probes and in situ detection reagents were purchased from Merck. PLA was performed following manufacturer’s instructions with minor modifications [57, 58]. After protein blocking, neurons were incubated with primary antibodies overnight at 4 °C: rabbit anti-MBD2 at 1:200 (#M7318, Sigma), mouse anti-PYK2 at 1:200 (#3480S, Cell Signaling), and chicken anti-MAP2 at 1:500 (#188006, Synaptic Systems). Samples were washed with a solution of 0.15 M NaCl, 0.01 M Tris, 0.05% Tween-20, at pH 7.4 (Buffer A), incubated with Mouse-minus and Rabbit-plus PLA probes (secondary antibodies labeled with complementary DNA strands) in Duolink antibody diluent for 1 h at 37 °C, and washed with Buffer A. The two DNA strands were enzymatically ligated, provided that they were in close proximity (< 30 nm) [58], for 30 min at 37 °C and another wash with Buffer A. This was followed by the enzymatic rolling-circle amplification of DNA and hybridization of Cy3-labelled oligonucleotides (PLA orange) for 100 min at 37 °C. Samples were then washed with a solution of 0.1 M NaCl and 0.2 M Tris, at pH 7.5. After the PLA process, samples were incubated with goat anti-chicken Alexa Fluor 488 (#A11039, Invitrogen, Waltham, MA, USA), donkey anti-mouse Alexa Fluor 594 (#A21203, Invitrogen), and donkey anti-rabbit Alexa Fluor 647 (#A31573, Invitrogen) secondary antibodies at 1:500 for 1 h at RT, followed by 10 min Hoechst incubation and PBS wash. Coverslips were mounted in glycerol and stored at 4 °C until imaging. Image acquisition was performed with Leica STELLARIS 5 confocal microscope.

### RNA preparation, sequencing and bioinformatics analysis

For sequencing of RNA in adult hippocampus, mice were killed by cervical dislocation, the brains were rapidly dissected, and the two hippocampi were dissected out in ice-cold Hanks’ buffered saline, snap-frozen in liquid nitrogen and stored at −80 °C. Tissue was disrupted with 700 µL QIAzol Lysis Reagent in 2 mL Dounce homogenizers, followed by syringe and needle homogenization and RNA extraction with RNeasy Mini Kit (Qiagen, #74104), following the manufacturer's instructions. Hippocampal cells in culture were treated with vehicle (MilliQ H_2_O) or 40 µM sodium glutamate (Sigma, #G1626) for 2 h at 37 °C and 5% CO_2_, after which the media was discarded, cells were rinsed twice with warm Dulbecco’s PBS and harvested on ice with 250 µL QIAzol Lysis Reagent per well. The lysate was snap-frozen in liquid nitrogen and stored at −80 °C. When all samples were harvested, lysates were brought back to room temperature and homogenized with syringe and needle. RNA extraction was as above. The RNA was quantified with a Nanodrop 1000 spectrophotometer, and its integrity checked with a Bionalyzer (Agilent RNA 6000 nano kit). RNA was retro-transcribed with SuperScript IV reverse transcriptase (Life Technologies). RNAseq was carried out at *iGenSeq core facility* (Genotyping and sequencing), at ICM (Paris Brain Institute). Reverse-transcribed mRNA (500 ng) was used for mRNA library preparation, realized following manufacturer’s recommendations (NEBnext Ultra 2 mRNA Kit from New England Biolabs). Final samples pooled library prep were sequenced on Nextseq 500 ILLUMINA with MidOutPut cartridge (2 × 130 million of 75-base reads) with 2 runs (4plex and 4plex), corresponding to 2 × 30 million reads per sample after demultiplexing.

The quality of the raw data was evaluated with FastQC. Poor quality sequences were trimmed or removed with Fastp software to retain only good quality paired reads. Star v2.5.3a [59] was used to align reads on mm10 reference genome using default parameters except for the maximum number of multiple alignments allowed for a read, which was set to 1. Quantification of gene and isoform abundances was done with RSEM 1.2.28 [60] on RefSeq catalog, prior to normalization with edgeR bioconductor package [61]. Differential analysis was conducted with DESeq2 [62]. Multiple hypothesis adjusted *p* values were calculated with the Benjamini-Hochberg procedure to control FDR. Enrichment analysis was conducted with clusterProfiler R package (v4.2.2) [63]. Gene ontologies and pathways analyses, and gene set enrichment analysis (GSEA) were done with Enrichr [64, 65] https://maayanlab.cloud/Enrichr/enrich.

### Pilocarpine treatment

Status epilepticus was induced with pilocarpine [31], as previously described [32]. Male PYK2- KO mice or wild-type littermates (10–12 week-old) were pretreated with lithium chloride LiCl (L4408, Sigma-Aldrich, 423 mg kg^−1^, i.p.) and methylscopolamine (1 mg kg^−1^, i.p., S2250, Sigma-Aldrich), 24 h and 30 min before pilocarpine, respectively. Pilocarpine (70 mg kg^−1^, i.p., P6503, Sigma-Aldrich). Control animals received lithium, methylscopolamine, and saline instead of pilocarpine. Three hours after status epilepticus onset, the mice (except the group of mice sacrificed at 20 min after SE) received a diazepam injection (10 mg kg^−1^, s.c.) in order to improve survival, decrease their anxiety and induce muscle relaxation. Brains were collected for immunofluorescence 20 min and 3 h after status epilepticus. Another batch of mice was kept for later analyses. During 3 days, they were kept warm by placing their cages on a warm plate (30 °C) and they received daily injections of glucose (50 g l^−1^, in saline, 0.3 ml, s.c.) and were fed with enriched food. They were subjected to the open field paradigm 7 days after status epilepticus and sacrificed 24 h later for Fluoro-Jade staining.

### Open field

To check spontaneous locomotor activity of mice 7 days after treatment with pilocarpine, we used the open field. Briefly, the apparatus consisted of a white square arena measuring 40 cm × 40 cm × 40 cm length-width-height. Dimly lit intensity was 60 lux throughout the arena. Animals were placed in the center of the arena and allowed to explore freely for 30 min. Spontaneous locomotor activity was measured. At the end of each trial, any defecation was removed, and the apparatus was wiped with 35% ethanol. Animals were tracked and recorded with SMART junior software (Panlab, Spain).

### Fluoro-Jade staining and counting

Eight days after pilocarpine treatment, mice treated with vehicle or pilocarpine were deeply anesthetized and immediately perfused transcardially with 4% paraformaldehyde/phosphate buffer. Brains were removed and post-fixed for 1–2 h in the same solution, cryoprotected by immersion in 30% sucrose/PBS and then frozen in dry-ice-cooled isopentane. Serial coronal cryostat sections (30-μm thick) were collected on superfrost slides. Sections were processed for Fluoro-Jade staining (Histo-Chem, Jefferson, AR, USA) as described [66]. Images from stained sections were captured with a Nikon DXM 1200 F digital camera attached to a Nikon Eclipse E600 light microscope (×40 oil objective). Cell density/field was counted in CA1, CA3 and DG hippocampal regions. We counted three slices per mouse (7 mice per genotype), and an average of the Fluoro-Jade-positive cell density was calculated for each region.

### Statistical analysis

For each experiment or condition, the sample numbers are reported in Supplementary Table 1. Statistical analyses were carried out using the GraphPad Prism 6.0 software. Data distribution was checked for normality with d’Agostino-Pearson and Shapiro-Wilk normality tests. If these tests detected a significant difference from normal distribution or if the number of samples was <7, statistical comparisons of three groups or more were done with Kruskall and Wallis test, followed by two-by-two comparisons with Dunn’s test, and two-group comparisons with the Mann-Whitney test. Normally distributed batches with seven samples or more were analyzed with two-way ANOVA followed by the Holm-Sidak test or two-group comparisons with Student’s *t* test. All two-by-two comparisons were two-tailed. Values of *p* < 0.05 were considered statistically significant. All statistical analyses results are presented in Supplementary Table 1.

## Supplementary information

Supplementary Figures

Supplementary Tables Legends

Supplementary Table 1: detailed

Supplementary Table 2: RNAseq differentially expressed genes between WT and PYK2-KO adult mouse hippocampus

Supplementary Table 3: Analysis with EnrichR of differentially expressed genes in hippocampus

Supplementary Table 4: RNAseq differentially expressed genes between WT and PYK2-KO embryonic mouse hippocampal neurons in culture

Supplementary Table 5: RNAseq differentially expressed genes in response to glutamate in WT and PYK2-KO embryonic mouse hippocampal neurons in culture

Supplementary Table 6: Analysis with EnrichR of differentially expressed genes in response to glutamate in WT and KO hippocampal neurons in culture

Original data for Fig. 5

## Data Availability

Data can be available on demand. Sequencing data are available at https://www.ncbi.nlm.nih.gov/geo (pending).
