# Supplementary material for: Glutamate-induced nuclear translocation of PYK2 in hippocampal neurons, interaction with MBD2, and role in cell death in a model of epilepsy
Source: Cell Death Dis. 2026 Apr 22;17(1):535. doi: 10.1038/s41419-026-08628-x (PMC13237024; doi:10.1038/s41419-026-08628-x)
Supplement: Supplementary file 1 — Supplementary Figures [file 41419_2026_8628_MOESM1_ESM.docx]

**Glutamate-induced nuclear translocation of PYK2 in hippocampal neurons, interaction with MBD2, and role in cell death in a model of epilepsy**

Albert Giralt, Tiago Mendes, Enrica Montalban, Carmen Cifuentes-Diaz, Marcos Galán-Ganga, Margot Chouchana, Benoît de Pins, Sophie Longueville, Damien Carrel, and Jean-Antoine Girault

**SUPPLEMENTARY FIGURES**

**Supplementary Figure 1: MBD2 coimmunoprecipitates with PYK2**

**A**) Interaction of PYK2 with MBD2 was analyzed by a co-immunoprecipitation assay in protein extracts from WT hippocampal neurons in culture (DIV20-21) treated with vehicle or glutamate (Glu, 40 µM for 15 min). We immunoprecipitated PYK2 protein with the rabbit anti-PYK2 antibody (**IP**) and rabbit immunoglobulins G (**IgG**) were used as a control. Membranes were then subjected to immunoblotting for PYK2 and MBD2 as indicated (see full length blots in **Supplementary Figure 1**). **B**) Densitometry quantification of MBD2 (PYK2/MBD2 association) results in IP samples as in **A**. Two-tailed Mann Whitney test not significant, n = 3-5 per group.

**Supplementary Figure 2: Translocation of MBD2 and PYK2 to the nucleus following glutamate treatment.**

Hippocampal neurons in culture were used for monitoring nuclear translocation of PYK2 and MBD2. Images correspond to the quantifications shown in Fig. 3A-B. **A**) WT and PYK2-KO neurons were treated with glutamate 40 µM for 15 min or vehicle (H_2_O). B) WT neurons were treated as in A in the presence of DMSO (vehicle for the inhibitors) or the indicated inhibitors, (MK801, 10 µM, nifedipine, 1 µM, or FK506, 1µM) added 30 min prior to glutamate. Immunostaining was done for MBD2 and PYK2 and nuclear staining with Hoechst 33342. Single confocal sections are shown, scale bar A, 10 µm, B 50 µm.


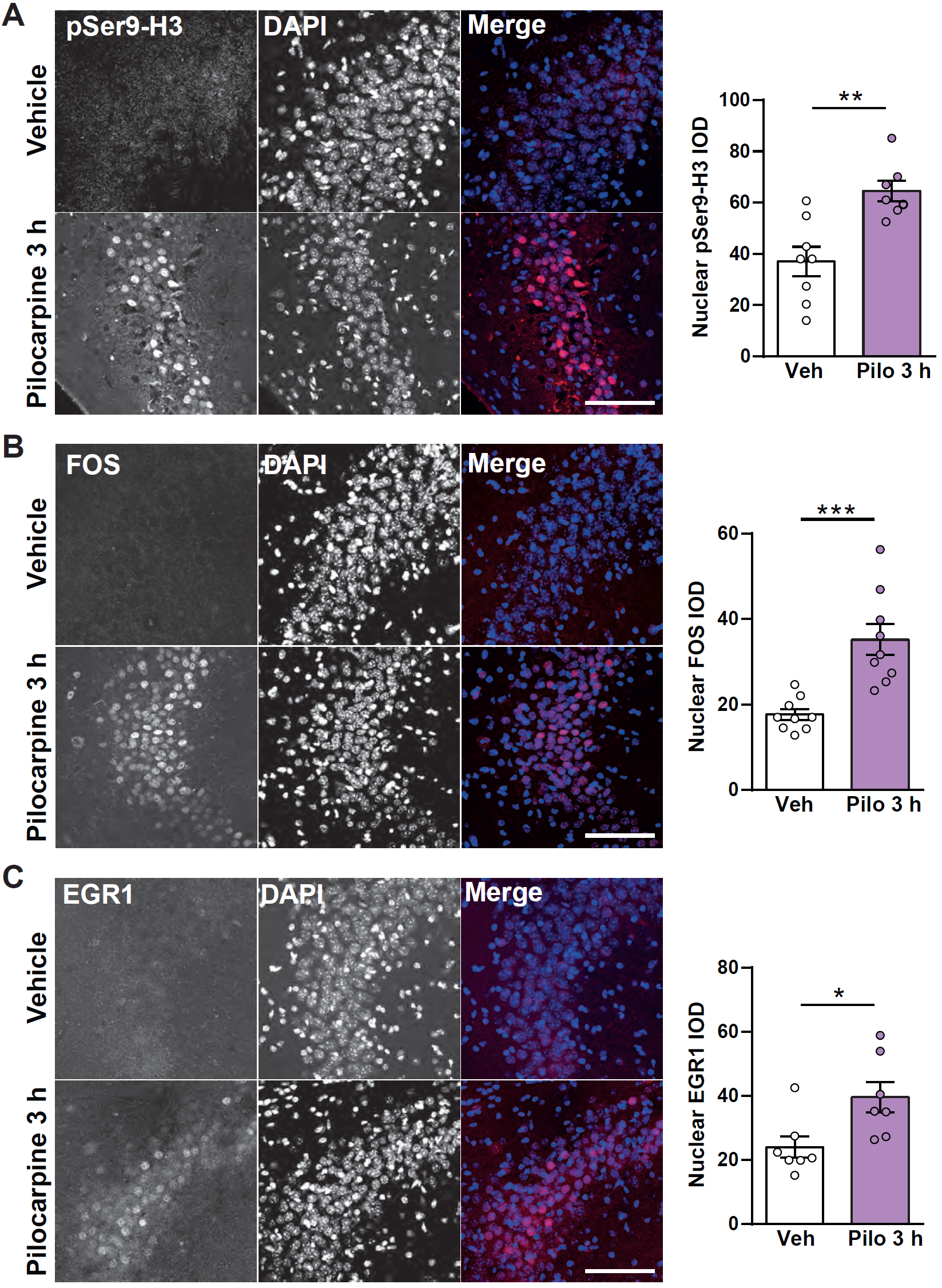


**Supplementary Figure 3: Pilocarpine-induced status epilepticus increases phosphorylation of histone H3 and nuclear immunofluorescence of immediate-early genes, FOS and EGR1**. Mice were treated with vehicle or lithium/pilocarpine as described in Fig. 7A and Material and Methods. Pilocarpine-treated mice were sacrificed 3 h after SE onset and control mice at a similar time after vehicle treatment. Immunostaining was carried out for (**A**) histone H3 phosphorylated on Ser9 (pSer9-H3), (**B)** FOS, and (**C**) EGR1, with DAPI nuclear staining. **Left side:** confocal sections in CA3 region. Scale bar, 100 µm. **Right side:** Quantification of nuclear IOD of immunofluorescence. Student’s t test, pSer9-H3, t_13_ = 3.82, p = 0.002, n = 8 for vehicle and 7 for pilocarpine, FOS, t_16_ = 4.56, p = 0.0003, n= 9 in each group, EGR1, Mann-Whitney test, p = 0.026, n = 9 in each group. *, p < 0.05, **, p < 0.01, *** p <0.001. See **Supplementary Table 1** for detailed statistical analyses.

**Supplementary Figure 4: PYK2-KO modifies the behavioral consequences of pilocarpine-induced status epilepticus.** Mice were placed in an open field a week after treatment with vehicle or lithium/pilocarpine (see **Fig. 8**). **A**) Quantification of time spent in arena center. Kruskal-Wallis' test, p < 0.0015, group comparison with Dunn's test, **, p <0.01, n, WT Veh, 6, WT Pilo, 7, KO Veh, 7, KO Pilo, 5. (**B**) Time course of parallel index. (**C**) Analysis of the 8 last min in E (green shaded area). Kruskal-Wallis' test, ns, group comparison with Dunn's test, * p < 0.035.
