# Supplementary material for: Glutamate-induced nuclear translocation of PYK2 in hippocampal neurons, interaction with MBD2, and role in cell death in a model of epilepsy
Source: Cell Death Dis. 2026 Apr 22;17(1):535. doi: 10.1038/s41419-026-08628-x (PMC13237024; doi:10.1038/s41419-026-08628-x)
Supplement: Supplementary file 2 — Supplementary Tables Legends [file 41419_2026_8628_MOESM2_ESM.docx]

**Glutamate-induced nuclear translocation of PYK2 in hippocampal neurons, interaction with MBD2,**

**and role in cell death in a model of epilepsy**

**Supplementary Tables Legends**

**Supplementary Table 1: Statistical analyses**

The tables report the detailed statistical analyses and results for the figures in which a quantitative comparison was done.

**- Figure 1B.** **PYK2 nuclear translocation in hippocampal neurons in culture treated for 15 min with vehicle or glutamate** (Glu, 40 µM) without or with MK801 (10 µM) or FK506 (1 µM), added 30 min before. A second set of cultured hippocampal neurons were treated for 15 min with vehicle or glutamate (Glu, 40 µM), placed in fresh medium and fixed 3 h later.

**- Figure 1D.** **Effects of PYK2 mutations on its nuclear translocation in hippocampal neurons in culture treated for 15 min with vehicle or glutamate** (Glu, 40 µM). Hippocampal neurons cultured for 20-21 DIV were transfected with plasmids coding for GFP fused to wild-type PYK2, PYK2_Y402F_, PYK2_1-840_, or PYK2_ΔNLS/NLS_.

**- Figure 2B.** **MBD2 nuclear translocation in hippocampal neurons in culture** treated for 15 min with vehicle or glutamate (Glu, 40 µM) without or with MK801 (10 µM) or FK506 (1 µM), added 30 min before. A second set of cultured hippocampal neurons were treated for 15 min with vehicle or glutamate (Glu, 40 µM), placed in fresh medium and fixed 3 h later.

**- Figure 2D.** **MBD2 nuclear translocation in WT and PYK2 KO hippocampal neurons** in culture treated for 15 min with vehicle or glutamate (Glu, 40 µM).

**- Figure 3A.** **Quantification of PYK2 nuclear localization in response to glutamate** 40 µM for 15 min and vehicle or various pharmacological inhibitors (MK801, 10 µM, nifedipine, 1 µM, or FK506, 1µM) added 30 min prior to glutamate, in conditions similar to those used below for PLA assays (see Figure 3D).

**- Figure 3B.** **Quantification of MBD2 nuclear localization in response to glutamate** 40 µM for 15 min and vehicle or various pharmacological inhibitors (MK801, 10 µM, nifedipine, 1 µM, or FK506, 1µM) added 30 min prior to glutamate, in conditions similar to those used below for PLA assays (see Figure 3D).

**- Figure 3 D. PYK2 MBD2 interactions evaluated by PLA.** Fixed neurons treated as in 3B were labeled with PYK2, MBD2, and MAP2 antibodies. The PLA reaction was carried out with secondary antibodies labeled with complementary DNA strands and amplified DNA detected with Cy3-labelled oligonucleotides. Quantification of results using the ratio of the PLA area over the cell area.

**Figures 4B-D.** **PYK2 and MBD2 intranuclear distribution and interaction investigated with electron microscopy.** WT hippocampal neurons were cultured for 20-21 DIV and treated for 15 min with vehicle or glutamate (Glu, 40 µM). (B) Quantification of PYK2-associated gold particles in the nucleoplasm and nucleolus/coiled bodies (CBs) in vehicle (Veh) or glutamate (Glu)-treated cells. (C) Quantification of MBD2 particles. (D) Quantification of PYK2/MBD2 gold particle clusters.

**- Figures 4G-I. Electron microscopy localization of MBD2 in hippocampal neurons in adult WT or PYK2 KO mice.** Dorsal hippocampus CA3 tissue from adult WT and PYK2-KO mice was immunogold-labeled for MBD2 (10-nm particles). (G) Quantification of the MBD2 particles density (number of particles per µm²) in the whole nucleus, expressed as a % of WT mean in the same experiments. (H) Quantification of MBD2 particles in nucleoplasm. (I) Quantification of MBD2 particles in nucleosomes/CBs.

**- Figure 5A-C. Selective alterations of histone modifications in the hippocampus of PYK2-KO mice. A**) Immunoblotting analysis of H1 monomethylated on lysine26 (**H1K26me1**) in hippocampal homogenates from WT and PYK2-KO mice. **B**) Immunoblot analyses for histone H4 acetylation on lysine 5 (**H4K5ac**) and lysine 8 (**H4K8ac**). **C**) Immunoblot analyses for histone H3 di/trimethylation on lysine 4 (**H3K4me2/3**), monomethylation on lysine 9 (**H3K9me**), acetylation on lysine 14 (**H3K14ac**) and lysine 18 (**H3K18ac**). Tubulin was used as a loading control. Values are expressed as % of the mean band IOD in WT.

**- Figure 7A. PYK2 translocation into the nucleus of hippocampal neurons upon pilocarpine treatment.** Adult WT mice were treated with lithium/pilocarpine and killed for histological examination 20 min or 3 h after *status epilepticus* (SE) onset and compared with vehicle-treated mice. The PYK2 immunofluorescence IOD intensity ratio between the nucleus and cytoplasm was calculated in CA1, CA3 and dentate gyrus (DG). Nuclei contours were determined by DAPI staining.

**- Figure 7B. MBD2 translocation into the nucleus of CA3 hippocampal neurons upon pilocarpine treatment.** Adult WT mice were treated with lithium/pilocarpine and killed for histological examination 20 min or 3 h after *status epilepticus* (SE) onset and compared with vehicle-treated mice. The MBD2 immunofluorescence IOD intensity ratio between the nucleus and cytoplasm was calculated in CA3. Nuclei contours were determined by DAPI staining.

**- Figure 8A. Effects of pilocarpine on time of first seizure and of status epilepticus onset in WT and PYK2-KO mice.** WT and PYK2-KO adult mice were subjected to lithium/pilocarpine treatment, visually monitored and the time to reach the first seizure and the SE onset were recorded.

**- Figure 8B-C. Locomotor activity of WT and PYK2-KO in an open field, 7 days after treatment with vehicle or lithium/pilocarpine.** Only mice who had displayed a SE were included. (B) Total locomotion during 30 min. (C) The same data as a time course in 1-min bins.

**- Figure 8E. Delayed effects of pilocarpine-induced status epilepticus in WT and PYK2 KO mice.** The day after open field (8 days after vehicle or lithium/pilocarpine treatment), WT and PYK2-KO mice were perfused and tissue used for immunohistofluorescence of synaptophysin and PSD-95. The number of PSD-95-positive, synaptophysin-positive puncta, and their colocalized clusters was quantified in vehicle-treated and pilocarpine-treated WT and PYK2-KO mice.

**- Figure 8G. PYK2-KO decreases the neurotoxic effects of pilocarpine-induced epilepsy in vivo.** Eight days after lithium/pilocarpine-induced status epilepticus fluoro-Jade-positive cells were counted in each hippocampal region of WT and PYK2-KO mice.

**- Figure 8H. PYK2-KO decreases the neurotoxic effects of glutamate in hippocampal neurons in culture.** Cultured hippocampal neurons from WT and PYK2-KO embryos were transfected at DIV 18-19 with plasmids coding for GFP or GFP fused to wild-type PYK2 or PYK2ΔNLS/NST (with mutations of nuclear addressing sequences). Two days later, neurons were treated with vehicle or glutamate (125 µM), fixed after 24 h, and imaged for GFP fluorescence. The number of GFP-positive neurons per slide was counted in vehicle and glutamate-treated cultures and expressed as a percentage of the number of GFP-positive neurons in control cultures treated with vehicle. The percentage of surviving GFP-positive neurons was analyzed.

**- Supplementary Figure 1: MBD2 coimmunoprecipitates with PYK2.**

**- Supplementary Figure 3: Pilocarpine-induced status epilepticus increases phosphorylation of histone H3 and nuclear immunofluorescence of immediate-early genes, FOS and EGR1**. Mice were treated with vehicle or lithium/pilocarpine and sacrificed 3 h after SE onset. Control mice were killed at a similar time after vehicle treatment. Immunostaining was carried out for histone H3 phosphorylated on Ser9 (pSer9-H3), FOS, and EGR1, and quantified in CA3.

**- Supplementary Figure 4A: PYK2-KO modifies the behavioral consequences of pilocarpine-induced status epilepticus.** Mice were placed in an open field a week after treatment with vehicle or lithium/pilocarpine. Quantification of time spent in arena center.

**- Supplementary Figure 4C: PYK2-KO modifies the behavioral consequences of pilocarpine-induced status epilepticus.** Mice were placed in an open field a week after treatment with vehicle or lithium/pilocarpine. Analysis of the 8 last min of the time course of parallel index (total recording 30 min).

**Supplementary Table 2: RNAseq in hippocampus from adult WT and PYK2-KO mice**

**a- All results**

**b- Selection of genes in (a) with PValue < 0.01**

**c- Selection of genes in (b) increased in PYK2 KO mice**

**d- Selection of genes in (b) decreased in PYK2 KO mice**

**e- Analysis specifics**

**Supplementary Table 3: EnrichR analysis of gene expression alterations by PYK2-KO (RNAseq) in adult hippocampus**

**a- KO effects on gene ontologies and pathways**

**b- Protein-protein interactions hub analysis in downregulated genes**

Gene ontologies and pathways analyses, and gene set enrichment analysis (GSEA) was done with Enrichr (refs. 64,65) <https://maayanlab.cloud/Enrichr/enrich>.

**Supplementary Table 4: RNAseq in WT and PYK2-KO hippocampal neurons in culture**

**a- All results**

**b- Selection of genes in (a) with PValue < 0.01**

**c- Selection of genes in (b) increased in PYK2 KO neurons**

**d- Selection of genes in (b) decreased in PYK2 KO neurons**

**e- Analysis specifics**

**Supplementary Table 5: Transcriptional analysis of glutamate treatment in WT and PYK2-KO hippocampal neurons in culture**

**a- All results of glutamate effects in WT cells**

**b- Selection of genes in (a) WT cells with PValue < 0.01**

**c- All results of glutamate effects in KO cells**

**d- Selection of genes in (c) KO cells with PValue < 0.01**

**e- Selection of genes in (b) increased by glutamate in WT cells**

**f- Selection of genes in (d) increased by glutamate in KO cells**

**g- Selection of genes in (b) decreased by glutamate in WT cells**

**h- Selection of genes in (d) decreased by glutamate in KO cells**

**i- Comparison of glutamate effects in WT and KO cells**

**j- Analysis specifics**

**Supplementary Table 6: EnrichR analysis of gene expression alterations by glutamate (RNAseq) in WT and PYK2-KO hippocampal neurons in culture**

**a- Glutamate upregulated genes ontologies and pathways**

**b- Glutamate downregulated genes ontologies and pathways**

**c- Protein-protein interactions hub analysis in genes downregulated by glutamate in WT neurons**

**c- Protein-protein interactions hub analysis in genes downregulated by glutamate in KO neurons**

Gene ontologies and pathways analyses, and gene set enrichment analysis (GSEA) was done with Enrichr (refs. 64,65) https://maayanlab.cloud/Enrichr/enrich.
