# Supplementary material for: Glutamate-induced nuclear translocation of PYK2 in hippocampal neurons, interaction with MBD2, and role in cell death in a model of epilepsy
Source: Cell Death Dis. 2026 Apr 22;17(1):535. doi: 10.1038/s41419-026-08628-x (PMC13237024; doi:10.1038/s41419-026-08628-x)
Supplement: Supplementary file 9 — Original data for Fig. 5 [file 41419_2026_8628_MOESM9_ESM.pdf]

ORIGINAL DATA for FIG 5

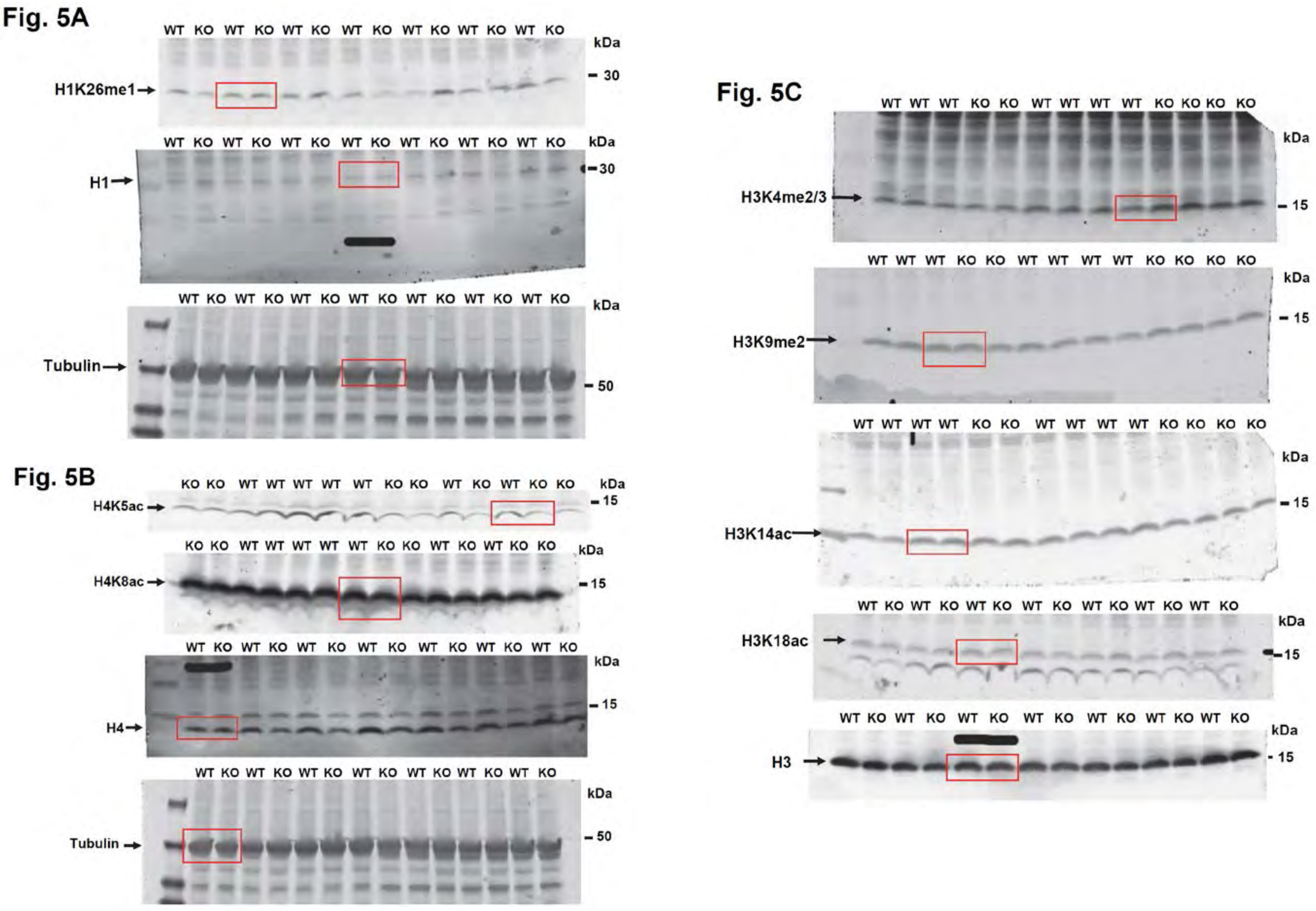

Supplementary Figure 5: original data for Figure 5: full length immunoblots of histone modifications. The various immunoblots correspond to Figures 5A, 5B and 5C as indicated. Note that the membranes were cut after transfer for incubation with various antibodies, and the full protein separation on electrophoresis gel is not available. Red boxes indicate regions shown in the corresponding Figure. Molecular weight markers positions are indicated in kDa.
